# Annexins A2 and A5 are potential early biomarkers of hepatocarcinogenesis

**DOI:** 10.1038/s41598-023-34117-8

**Published:** 2023-04-28

**Authors:** Ema Elvira Herrera-López, Dafne Guerrero-Escalera, Isaac Aguirre-Maldonado, Arely López-Hernández, Hilda Montero, María Angélica Gutiérrez‐Nava, Luis del Pozo-Yauner, Jaime Arellanes-Robledo, Javier Camacho, Julio Isael Pérez-Carreón

**Affiliations:** 1 Laboratorio de Enfermedades Hepáticas, Instituto Nacional de Medicina Genómica, Periférico Sur No. 4809, Col. Arenal Tepepan, Alcaldía Tlalpan, D.F., 14610 Mexico City, Mexico; 2 Departamento de Farmacología, Centro de Investigación y de Estudios Avanzados del IPN, Avenida Instituto Politécnico Nacional 2508, 07360 Mexico City, Mexico; 3 Instituto de Salud Pública, Universidad Veracruzana, Veracruz, Mexico; 4 División de Ciencias Biológicas y de la Salud, Departamento de Sistemas Biológicos, Universidad Autónoma Metropolitana-Xochimilco, Mexico City, Mexico; 5 Department of Pathology, College of Medicine, University of South Alabama, Alabama, USA; 6 Dirección de Cátedras, Consejo Nacional de Ciencia y Tecnología, Mexico City, Mexico

**Keywords:** Cancer models, Gastrointestinal cancer, Tumour biomarkers

## Abstract

Hepatocellular carcinoma (HCC) is a highly lethal liver cancer with late diagnosis; therefore, the identification of new early biomarkers could help reduce mortality. We determine the tissue and plasma status of five annexins during hepatocarcinogenesis by diethylnitrosamine-induced cirrhosis-HCC. We found that *Anxa5* was the earliest upregulated gene at week 12 after HCC initiation, while *Anxa1* and *Anxa2* were upregulated in advanced HCC stages (weeks 18 and 22). Furthermore, the protein level of Annexin A1, A2, A5 and A10 was increased from the early stages. Immunofluorescence and subcellular fractionation revealed Annexin A1, A2, and A5 in the cytoplasm and nuclei of tumor cells. Notably, increased plasma levels of Annexin A5 significantly (r^2^ = 0.8203) correlated with Annexin A5 levels in liver tissue from week 12 and gradually increased until week 22. Using the TCGA database, we found that the expression of *ANXA2* (HR = 1.7, *p* = 0.0046) and *ANXA5* (HR = 1.8, *p* = 0.00077) was associated with poor survival in HCC patients. In conclusion, we have identified Annexin A1 and A5 as potentially useful early biomarkers for poor prognosis in HCC patients.

## Introduction

Liver cancer is the fourth most frequent cause of cancer-related death and ranks seventh in cancer incidence worldwide^1^. Hepatocellular carcinoma (HCC) is the most common form of primary liver cancer and is frequently preceded by cirrhosis. Unfortunately, only 18% of patients bearing HCC have a 5-year survival rate, which places it as the second most lethal cancer, preceded only pancreatic cancer^2^. The lack of early biomarkers and the asymptomatic nature of HCC are some of the primary reasons for its late diagnosis; thus, when it is detected, most of the patients have a poor prognosis, limited treatment options, and a survival rate lower than 13 months^2,3^.

Viral infections, particularly HBV and HCV, remain the primary known cause of HCC, accounting for a significant proportion of cases worldwide. Despite the different nature between hepatitis viruses and chemical carcinogens, they may share similar mechanisms driving HCC development, such as chronic liver damage, persistent liver inflammation, intracellular oxidative stress, and deregulation of cell signaling pathways^4,5^. All these molecular perturbations contribute to cirrhosis, a major risk factor for HCC development.

Detection of serum alpha-fetoprotein (AFP) has been the most frequent diagnosis test mainly because it is inexpensive and noninvasive; however, it lacks sensitivity and specificity^6^. Since no ideal biomarkers have been found so far, identifying serum markers for early HCC diagnosis, monitoring the disease progression, treatment responsiveness, recurrence, and survival, remains an unmet need.

A rat hepatocarcinogenesis model induced with diethylnitrosamine (DEN) has been highly effective in recapitulating cirrhosis along with heterogeneous multinodular HCC in 18 weeks^7,8^. The alkylating capability of DEN causes chronic liver damage by chronologically inducing inflammation and fibrosis associated with cirrhosis and HCC, closely resembling the HCC development in humans^9,10^.

The DEN-induced HCC model produces chronic liver disease, including high cell turnover associated with cell death and proliferation, fibrosis that precedes cirrhosis, and unequivocal multi-tumor HCC development. However, a limitation of this hepatocarcinogen protocol for clinical application is that it does not aim to model the different etiologies identified for HCC development in humans, among which infection by different hepatitis viruses stands out. Despite the etiology, chronic liver damage is a common mechanism that may provide insight into molecular alterations in liver carcinogenesis^11^.

Annexins A1, A2, A5, A8, and A10 belong to the A family of annexins that are Ca^2+^-regulated phospholipid-binding proteins. Members of this family can be peripherally coupled to the surface of negatively charged membranes when they are calcium-linked. This capability provides them with membrane-bound functions, such as organization, transport, and interaction with the actin cytoskeleton^12^.

Using the cirrhosis-HCC model mentioned above, we have already reported a gene expression profile revealing increased expression of some annexin genes, including *Anxa2*^13^. Moreover, recent research has also found and proposed annexin A2 as a potential serum marker for patients with HCC^14^. Notably, some other annexins have the potential to be released into the bloodstream, but this phenomenon has not been addressed yet^15–18^.

Therefore, here we aimed to determine the status of some annexin family members at the gene and protein expression levels in liver tissue, as well as their plasma presence during experimental HCC progression. Besides, we analyzed the association of some annexin members with HCC patient survival using the TCGA database. This study suggests potential noninvasive markers for the early diagnosis of HCC.

## Results

### Features of experimental HCC progression

Multifocal HCC nodules and fibrosis after chronic administration of DEN were observed in livers (Supplementary Fig. S1b,c), consistent with previous reports^7^. We use the γ–glutamyl transferase (GGT) histochemistry as a tumor marker in liver sections^19^ GGT-positive foci were detected from W6, and the number and size increased over time, reaching up to 30% of the liver tissue (Supplementary Fig. S1d). Livers from W6 showed mild cell infiltration and ballooned hepatocytes, which increased up to W22 (16 weeks of DEN administration followed by a 6-week carcinogen-free period). Malignant nodules show dyschromatic appearance, well-defined edges, and loss of the liver parenchyma architecture. Furthermore, tumors > 3 mm in diameter were found at W22, in contrast to those observed at W18, which were ˂ 3 mm. Cellular and nuclear pleomorphism, such as anisokaryosis, macrokaryosis, and macronucleoli, were also noted as previously described for liver cancer^20^.

### The expression of annexin genes is differential during HCC progression

**Figure 1:**
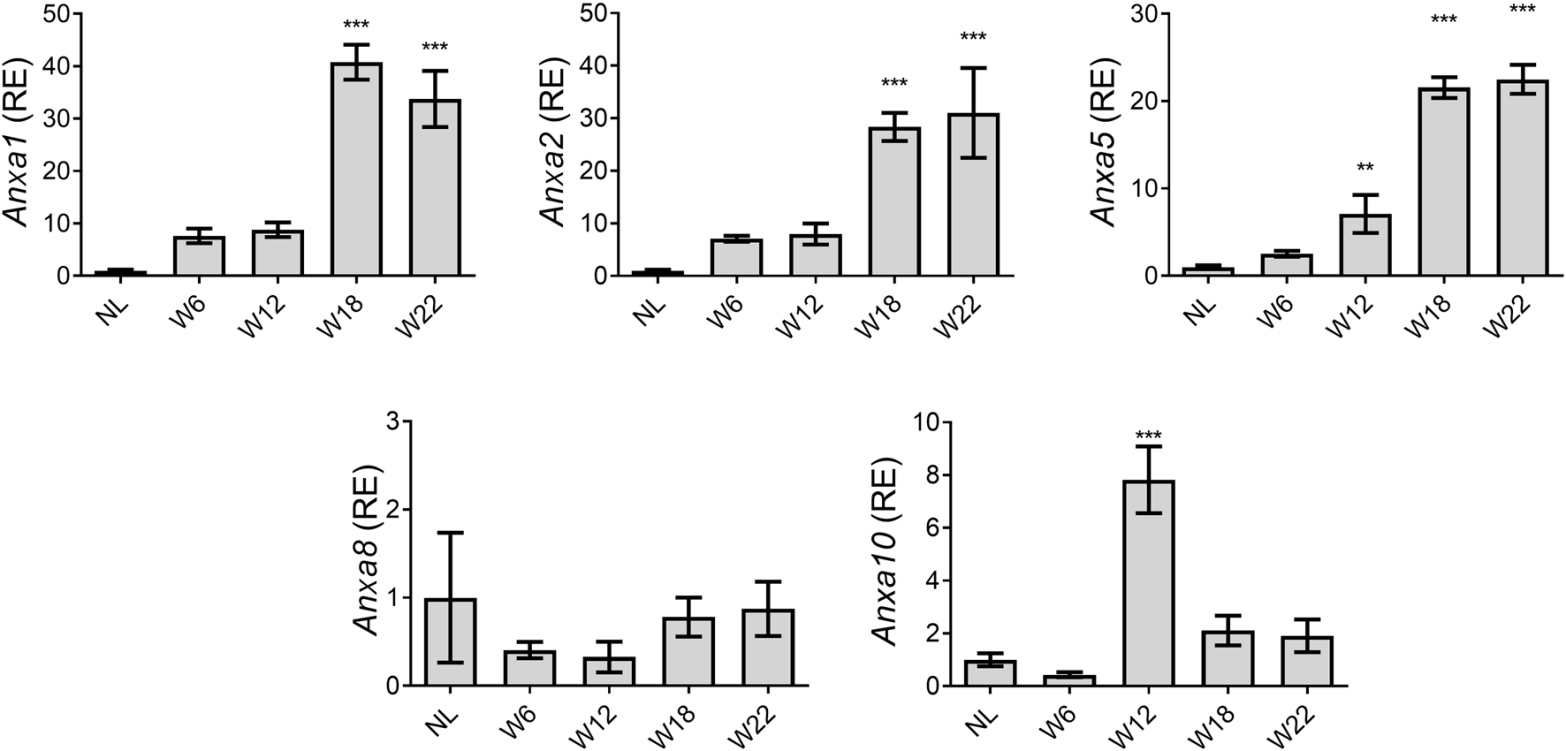
Expression of Anxa1, Anxa2, Anxa5, Anxa8, and Anxa10 genes during rat hepatocarcinogenesis. The relative mRNA levels of annexin genes in liver tissue were determined by RT-qPCR. *Anxa1, Anxa2, Anxa5, Anxa8*, and *Anxa10* expression was normalized to ribosomal 18 s mRNA level. Bars show the relative expression (RE) compared to the NL group and represent the mean ± SD. *p* values were calculated by the ANOVA test. n = 5 animals/group. *NL* normal liver, *W* week. ***p < 0.001; **p < 0.01.

The mRNA expression of the *Anxa1*, *Anxa2*, *Anxa5*, *Anxa8,* and *Anxa10* genes was analyzed by RT-qPCR to investigate their status during HCC progression. As shown in Fig. 1, the mRNA expression of *Anxa1*, *Anxa2,* and *Anxa5* genes was more than 20-fold higher (*p* < 0.001) in W18 and W22 groups as compared with that in the NL group. Furthermore, the mRNA expression of *Anxa2* and *Anxa5* genes constantly increased until W22*.* On the other hand, the mRNA expression of *Anxa10* gene transiently increased at W12 but decreased in the subsequent evaluated weeks (Fig. 1). In contrast, *Anxa8* mRNA expression levels during HCC progression were not different. Of note, the expression of *Anxa5* was the earliest increased by showing a sevenfold change (*p* < 0.01) at W12 as compared with NL group.

### Protein level of ANXA1, ANXA2, and ANXA5 progressively increases during hepatocarcinogenesis

**Figure 2:**
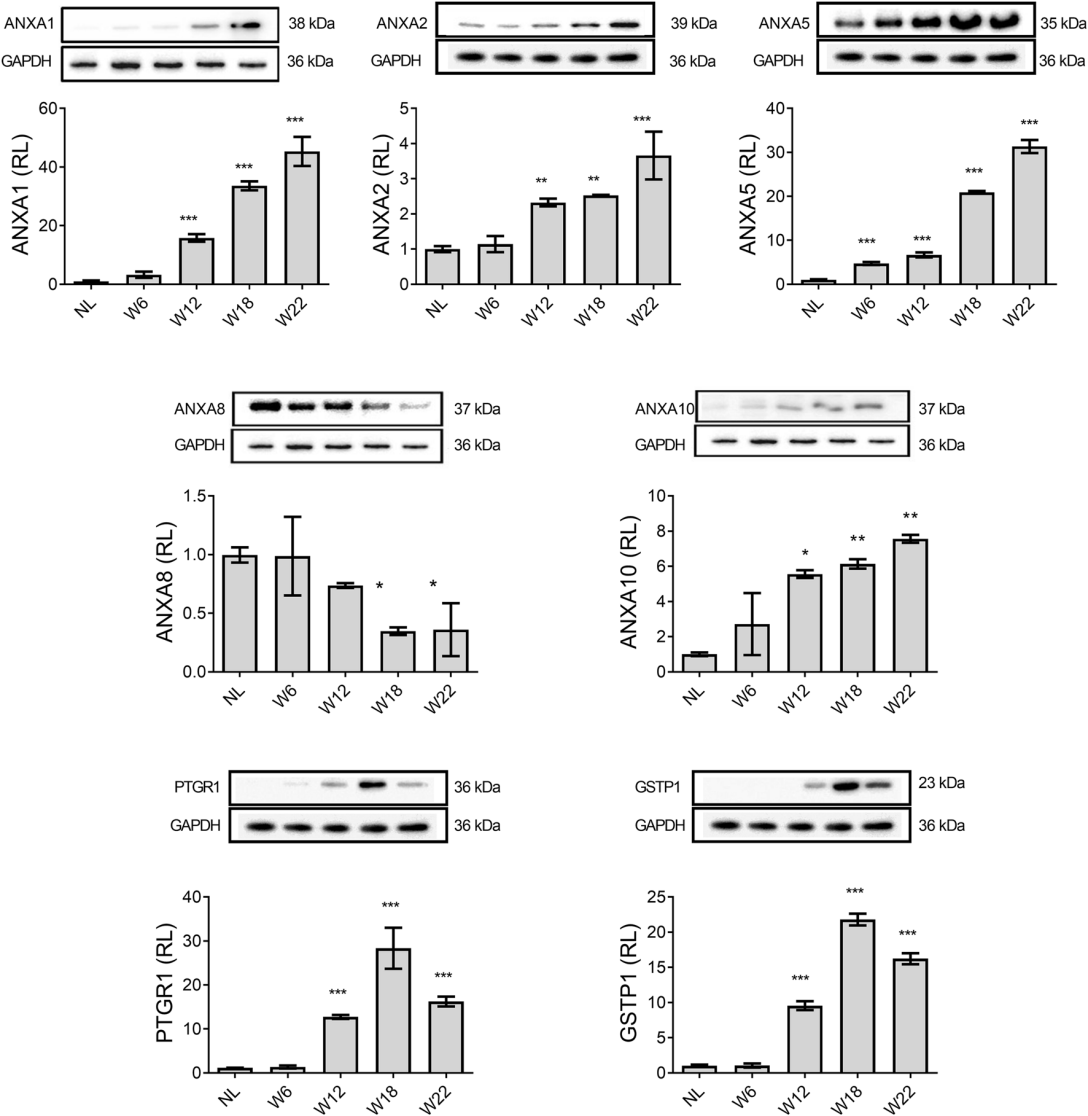
The protein level of annexins and well-known HCC markers. Representative images of Western blot analysis from liver tissue. Bars show the relative levels compared to the NL group and represent the mean ± SD. p values were calculated by ANOVA test. n = 5 animals/group. *NL* normal liver, *W* week. ***p < 0.001; **p < 0.01; *p < 0.05. Images were captured with the Uvitec MINI HD6 photo-documentation system. Original blots of figure are presented in Supplementary Information S1.

The protein level of ANXA1, ANXA2, ANXA5, ANXA8, and ANXA10, as well as of PTGR1 and GSTP1, two well-known liver tumor markers^8,21–26^, were determined from liver tissue by WB analyses. As shown in Fig. 2, ANXA1, PTGR1, and GSTP1 were not detected in NL samples. The proteins ANXA1, ANXA2, and ANXA10 showed a slight increase at W6 but significantly increased from W12 to W22 (*p* < 0.05), which correlated with the HCC progression. Interestingly, the level of ANXA5 gradually increased from W6 to W22 (*p* < 0.001), with a more significant increase at week 22, while PTGR1 and GSTP1 markers showed their highest levels at W18. Notably, a significant *(p* < 0.001*)* decrease in ANXA8 level was found at W18 and W22 as compared with NL samples (Fig. 2).

### ANXA1, ANXA2, and ANXA5 proteins are preferentially increased within cells of early nodules and HCC

**Figure 3:**
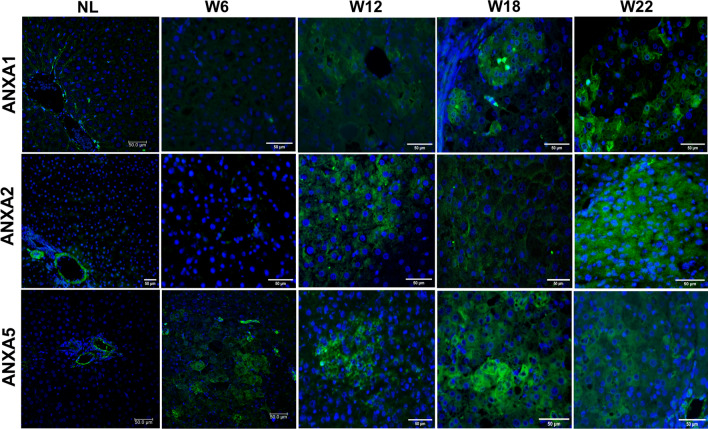
Increased annexins in cells of nodules during hepatocarcinogenesis. Representative images of ANXA1, ANXA2, and ANXA5 expression in NL tissue, preneoplastic lesions (W6 and W12), and HCC tumor cells (W18 and W22) were obtained by IF analysis. Annexin proteins were labeled with Alexa Fluor 488 (green), while nuclei were stained with DAPI (blue). Magnification: 400X. Scale bar = 50 μm. n = 5 animals/group. Images were captured with ZEISS Axio-A1 Microscopy.

Since ANXA1, ANXA2, and ANXA5 showed a continuous increment at both mRNA and protein levels, we selected them to analyze their histological and cellular localization during HCC progression. IF analysis revealed an increased number of ANXA1-, ANXA2-, and ANXA5-positive cells during HCC progression (Fig. 3). The highest presence of these proteins was observed at W22 and preferentially within HCC than in surrounding tissue. Cytoplasmic ANXA1 was detected at W12, W18, and W22. ANXA2 was only detected in central and portal veins in NL tissue, but its cytoplasmic localization increased in altered hepatocytes from W12 to W22. ANXA5 was observed in endothelial cells of NL tissue; in contrast, it was localized in altered hepatocyte foci from early hepatocarcinogenesis (W6), but a strong label was detected within nodules of W12, as well as in tumors of W18 and W22 tissues data not shown. This evidence indicates that the protein level of ANXA1, ANXA2, and ANXA5 is preferentially localized into altered cells during HCC progression and suggests that they might be helpful as HCC biomarkers.

### ANXA1, ANXA2, and ANXA5 are differentially compartmentalized in both nuclei and cytoplasm of altered liver cells

**Figure 4:**
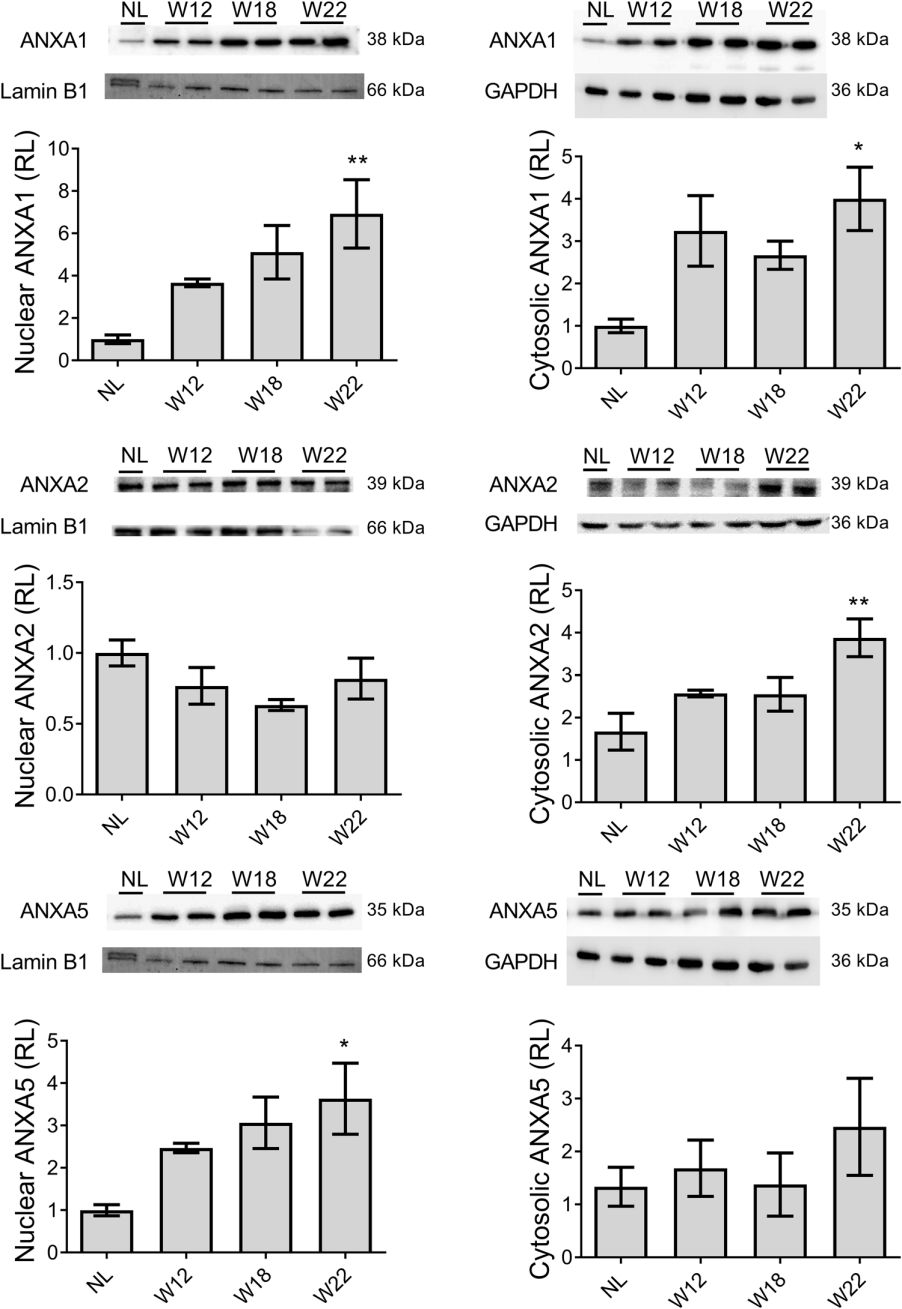
Subcellular expression levels of ANXA1, ANXA2, and ANXA5 proteins. The graphs show nuclear and cytosolic protein expression in liver samples from all experimental groups normalized to Lamin B1 and GAPDH, respectively. Bars show the relative levels (RL) compared to the NL group and represent the mean ± SD. p values were calculated by the ANOVA test. n = 3 animals/group. *NL* normal liver, *W* week. **p < 0.01; *p < 0.05. Images were captured with the Uvitec MINI HD6 photo-documentation system. Original blots of figure are presented in Supplementary Information S1.

Then, the subcellular expression levels of ANXA1, ANXA2, and ANXA5 during HCC progression were determined in nuclear and cytosolic fractions by WB analysis (Fig. 4). The level of these annexins tended to increase either in the nuclei or cytoplasm during hepatocarcinogenesis. For example, ANXA1 showed the highest level at W22 by reaching more than sevenfold (*p* < 0.01) and more than fourfold (*p* < 0.05) in the nuclei and cytoplasm, respectively, as compared with NL samples. On the other hand, ANXA2 showed no significant changes in the nuclei but significantly increased (fourfold, *p* < 0.01) in the cytoplasm at W22. Interestingly, ANXA5 significantly increased (> threefold, *p* < 0.05) in the nuclei at W22 as compared with NL samples (Fig. 4).

### Increased ANXA5 level in plasma correlates with liver tissue level during HCC progression

**Figure 5:**
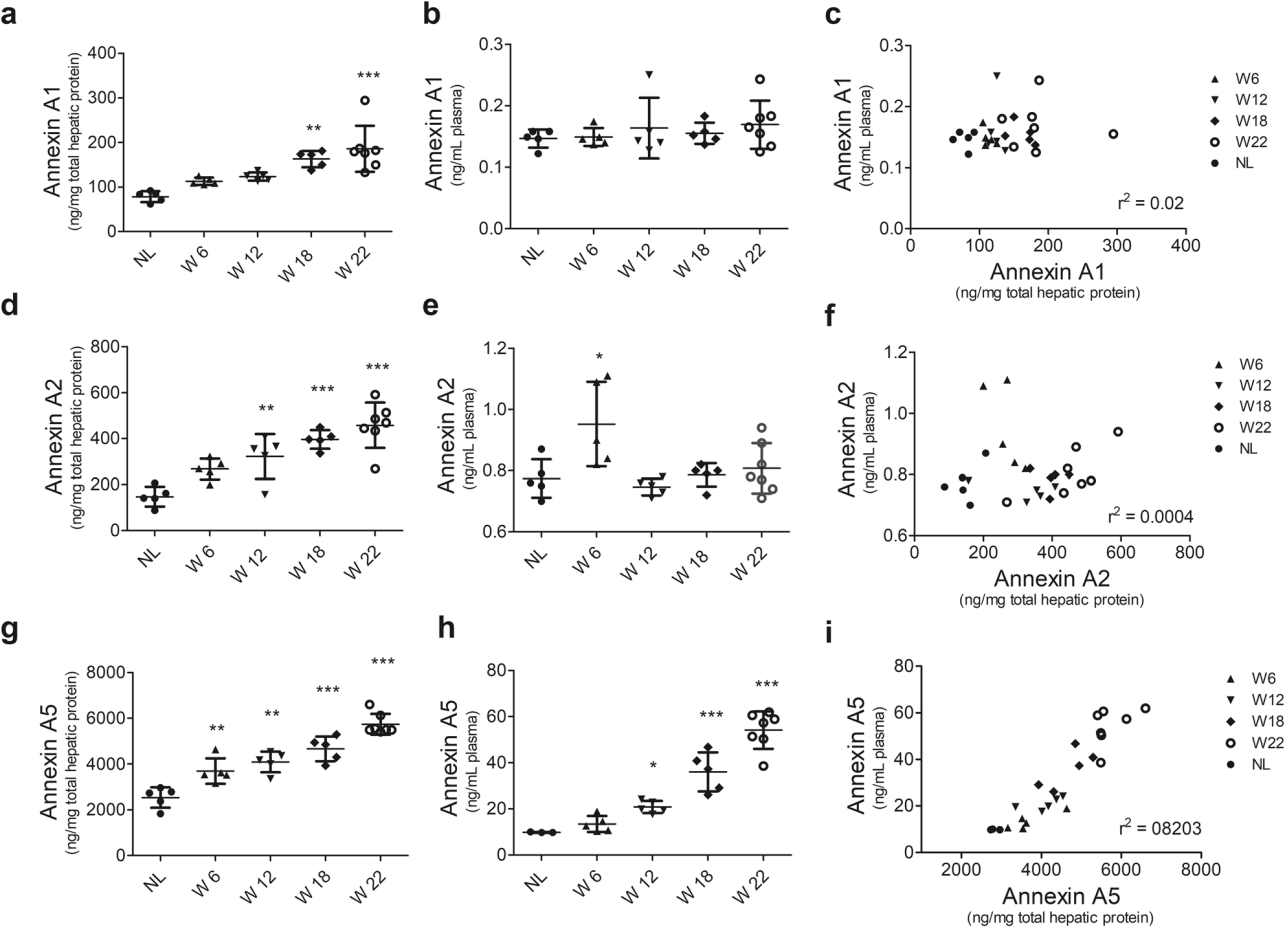
Liver and plasma annexin levels and their correlation during HCC progression. Determination of annexin levels by ELISA in liver tissues (**a**,**d**,**g**) or plasma samples (**b**,**e**,**h**). Correlation between the levels of annexin proteins in liver tissues and plasma samples (**c**,**f**,**i**). Data represent the mean ± SD. *p* values were calculated by the ANOVA test. n = 5 animals/group. *NL* normal liver, *W* week. ***p < 0.001; **p < 0.01; *p < 0.05.

Then, we compared the circulating levels of annexins in plasma with those in the liver tissue by ELISA. ANXA1 reached more than 150 ng/mg of protein (*p* < 0.01) in W18 and W22 groups (Fig. 5a); however, its plasma level neither changed nor correlated with that in the liver tissue (Fig. 5b,c). Increased concentration (*p* < 0.01) of ANXA2 in liver tissue was increased in W12, W18, and W22 (Fig. 5d), but similar to ANXA1, its level did not correlate with that in plasma (Fig. 5e,f). Of note, the concentration of ANXA5 in the liver tissue was more than ten times compared with that of ANXA1 and ANXA2 proteins. In the liver tissue, ANXA5 reached more than 3000 ng/mg of protein (*p* < 0.01) from the early HCC stage (W6) and progressively increased to 6000 ng/mg until the late HCC stage, namely W22 (Fig. 5g). Notably, a significant increment (*p* < 0.001) of ANXA5 in the plasma was detected from W12 that gradually increased until W22 of HCC progression (Fig. 5h). In addition, plasma ANXA5 level strongly correlated (r^2^ = 0.8203) with that in the liver tissue (Fig. 5i). This result indicates that the protein level of ANXA5 increases in the plasma alongside HCC tumorigenesis and may be a molecular candidate for early detection of HCC.

### ANXA5 is a significant prognostic value in human liver cancer

**Figure 6:**
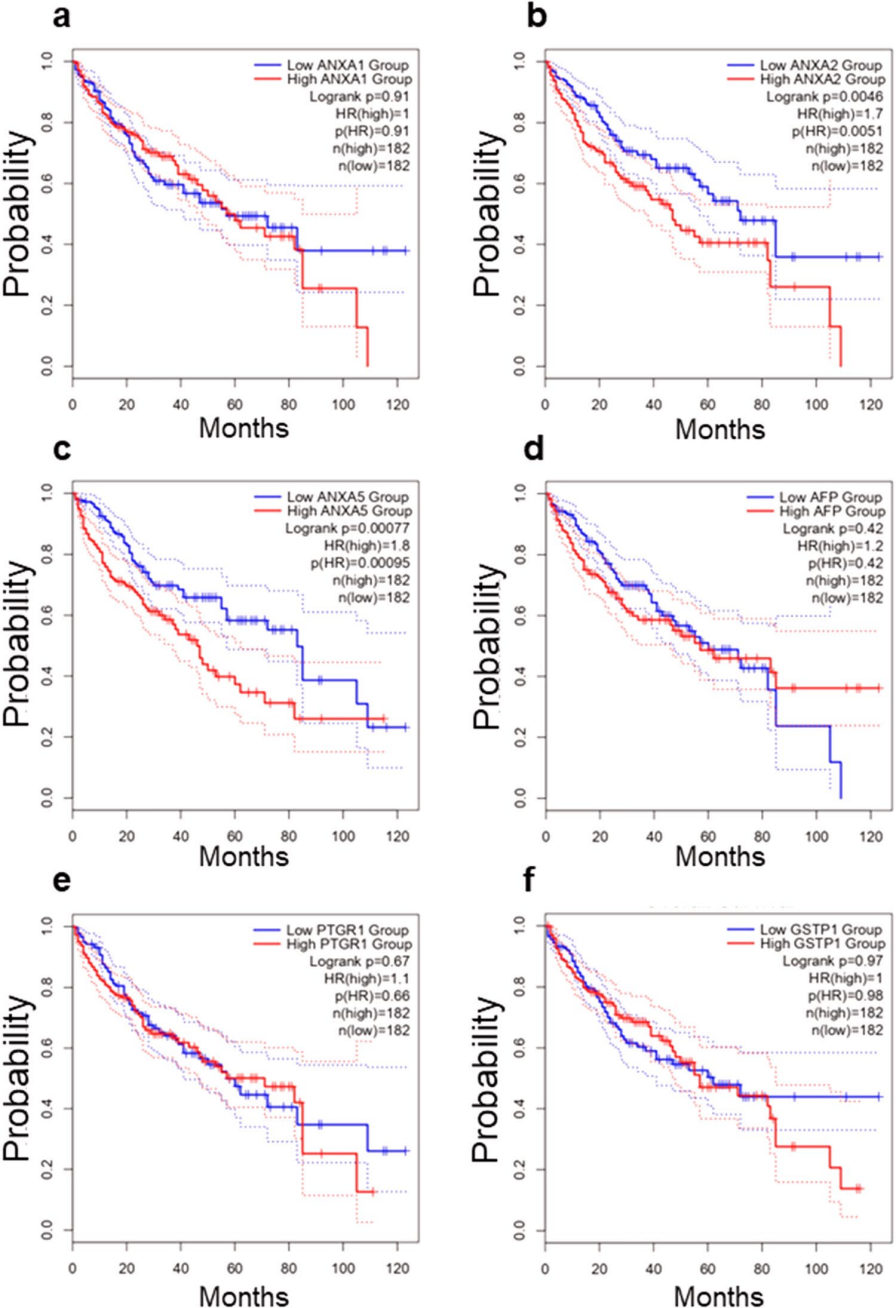
Kaplan–Meier curves for overall survival (OS). Plots show the percent of 5-year overall survival associated with the expression levels of *ANXA1* (**a**), *ANXA2* (**b**), *ANXA5* (**c**), *AFP* (**d**), *PTGR1* (**e**), and *GSTP1* (**f**) in HCC patients. Data were obtained from the liver hepatocellular carcinoma (LIHC) project, which comprises data from 364 HCC primary tumors, (https://portal.gdc.cancer.gov/projects/TCGA-LIHC).

Using the Liver Hepatocellular Carcinoma (LIHC) TCGA RNA-seq database, we determined the association between survival and mRNA expression of *ANXA1*, *ANXA2*, and *ANXA5* to validate their prognostic relevance in liver cancer, as well as that of *PTGR1*, *GSTP1*, two well-known liver tumor markers, and alpha-fetoprotein (AFP), widely used as a marker for the diagnosis of liver cancer in clinical practice^28^, to validate its potential prognostic relevance in liver cancer (Fig. 6). The analysis revealed that both the expression of *ANXA2* (HR = 1.7, *p* = 0.0046) and *ANXA5* (HR = 1.8, *p* = 0.00077) were associated with poor survival (Fig. 6b,c). In contrast, the expression of *ANXA1* (HR = 1, *p* = 0.91) was not associated with a worse prognosis (Fig. 6a). The expression of *AFP* (Fig. 6d) was not significant (HR = 1.2, *p* = 0.42). On the other hand, the expression of *PTGR1* (Fig. 6e), an enzyme reported as an HCC marker and capable of promoting cell proliferation and oxidative stress resistance in experimental HCC^29^, as well as that of *GSTP1* (Fig. 6f), an enzyme expressed in early stages of the rat HCC^30^, was not associated with worse prediction of survival (HR = 1.1, *p* = 0.67 and HR = 1, *p* = 0.97, respectively).

## Discussion

Based on a well-accepted animal model of diethylnitrosamine-induced cirrhosis-HCC and using available data in the LIHC-TCGA RNA-seq database, we have revealed the tissue and plasma status of five annexin family members during hepatocarcinogenesis and determined their usefulness as potential noninvasive markers for the diagnosis of HCC early stages. Some members of the annexins have been identified as circulating biomarkers in several types of cancer^22,24,26,31,32^

Our results show that ANXA1, ANXA2, ANXA5, and ANXA10 proteins increased alongside HCC progression from early stages and were sustained until week 22. Only ANXA5 significantly increased from week 6 (Fig. 2). For ANXA1, ANXA2, and ANXA5, the highest levels were detected at week 22, which was closely associated with the HCC progression. In contrast, the previously described tumor marker proteins GSTP1 and PTGR1^8^ decline at week 22 of HCC progression. While these two enzymes are involved in xenobiotic metabolism^31^ and their active status depends on the carcinogen presence or activity, the increased levels of annexins, even at week 22, when DEN was no longer administered, strongly suggest that the expression of annexins alongside the progression of HCC is a molecular alteration inherent to the transformation process of cancer cells but independent of the carcinogen presence, an ideal phenomenon when looking for markers directly associated to a disease and not to the experimental procedure used to induce it.

The ANXA1 was observed in both tumor and non-parenchyma cells, such as lymphocytes in tissues from week 12 to week 22. In addition, it has been reported that ANXA1 is released from immune cells, such as neutrophils activating monocytes and lymphocytes in a paracrine manner^33,34^. The role of ANXA1 has been controversial since it is unclear whether it promotes or inhibits cancer^33,35,36^. However, recent studies have reported that ANXA1 is an essential factor in inflammation and tumorigenesis^33,37^, liver regeneration^38^, and as a regulator of the immune response in cancer^34^.

ANXA2 is the member of the annexin family most studied as a circulating biomarker^14,22,31,32^ or tumor marker^39^ in various types of cancer; moreover, this protein has been widely considered a circulating biomarker for identifying early stages of liver cancer^14,21,22,24^. Our results demonstrate that ANXA2 increased at both mRNA and protein levels in the liver tissue as early as 12 weeks and persisted until 22 weeks during hepatocarcinogenesis (Figs. 1, 2, 5). Although we did not detect increased levels of this protein in the plasma of our animal model using ELISA, the elevated expression of the ANXA2 gene in the liver and previous clinical evidence suggest that it could be an early HCC biomarker. Circulating ANXA2 has been shown to have greater sensitivity, specificity, and predictive values in clinical cases of HCC than other well-accepted markers, such as AFP^14,22^.

Our results also show that protein levels of ANXA5 were strongly increased in liver tissue alongside the HCC progression. This significantly correlated with plasma levels (Fig. 5), suggesting that the ANXA5 detected in blood was likely released by liver tumor cells. Similarly, Serag and Elsayed have found that serum ANXA5 has a prognostic value for HCC in hepatitis C virus-associated cirrhosis patients^40^. These data reinforce the notion that the alteration of this protein during liver injury processes is closely linked to the transformation process of cancer cells but not to the etiological factor. Together, the above information supports and places ANXA5 as a useful circulating biomarker for the early diagnosis and prognosis of HCC.

The role of ANXA5 in liver carcinogenesis is unclear; nevertheless, at the intracellular level, ANXA5 has been linked to lymph node metastases in HCC through the mitogen-activated protein kinase and integrin extracellular signal-regulated kinase pathway. In addition, ANXA5 is known to recognize phosphatidylserine (PS) on the internal face of plasma membranes. However, when PS is present on the external side of the membrane, it becomes a signal for cellular phagocytosis and apoptosis. Interestingly, cancer cells exhibit PS on the outer plasma membrane without undergoing apoptosis, allowing them to evade phagocytosis. Although the molecular mechanism underlying this phenomenon is not yet fully understood, PS has been suggested as a target for a system that can deliver anticancer prodrugs using the specificity of ANXA5^41^.

Through microscopy, we have evidenced that ANXA1, ANXA2, and ANXA5 proteins are localized in both the nuclei and cytoplasm of preneoplastic cells and HCC tumors at week 22. We corroborated this subcellular localization by isolating nuclear and cytosolic protein fractions from liver tissues. Previous studies have reported the presence of ANXA2 in the nucleus. Specifically, Madureira et al. has demonstrated that exposure to genotoxic agents such as gamma radiation, UV radiation, and chromium VI can trigger ANXA2 translocation to the nucleus. These findings suggest that ANXA2 may play a role in DNA damage mitigation, as ANXA2 accumulation is impeded by antioxidant agents and stimulated by hydrogen peroxide, which are known to be related to reactive oxygen species^42^. Although ANXA2 is usually localized in the cytoplasm and cell membrane, we observed its nuclear localization in hepatocytes, which was probably induced by the genotoxic effect of DEN. Previous studies have shown that the nuclear localization of ANXA5 is controlled by signaling pathways involving serum tyrosine kinase factors^43^ and oxidative stress^44^. Therefore, this intriguing observation encourages investigating whether the role of ANXA2, and ANXA5 in the nucleus of cancer cells, either contribute to carcinogenesis or is associated with an anticancer mechanism.

Our evidence shows that the rat gene expressions of *Anxa1*, *Anxa2*, and *Anxa5* increased from the early stages of experimental model HCC and are closely associated with disease progression. From these, *ANXA2* and *ANXA5* human genes are significantly associated with the overall survival (OS) of HCC patients. Congruently, the relationship of annexins with OS has been previously documented^25^. Contrastingly, *GSTP1* and *PTGR1*, two well-known experimental HCC markers, and AFP, a serological protein widely used as a clinical HCC marker, did not show significant OS (Fig. 6). Our findings reveal that high expression of *ANXA2* and *ANXA5* correlates with poor survival in HCC patients with a better predictive value than AFP. On the other hand, we detected a significantly increased expression of ANXA1, ANXA2, and ANXA5 during the early HCC progression in the animal model. Also, we found a strong positive correlation of ANXA5 protein levels between plasma samples and liver extracts in the hepatocarcinogenesis model.

## Conclusion

**Figure 7:**
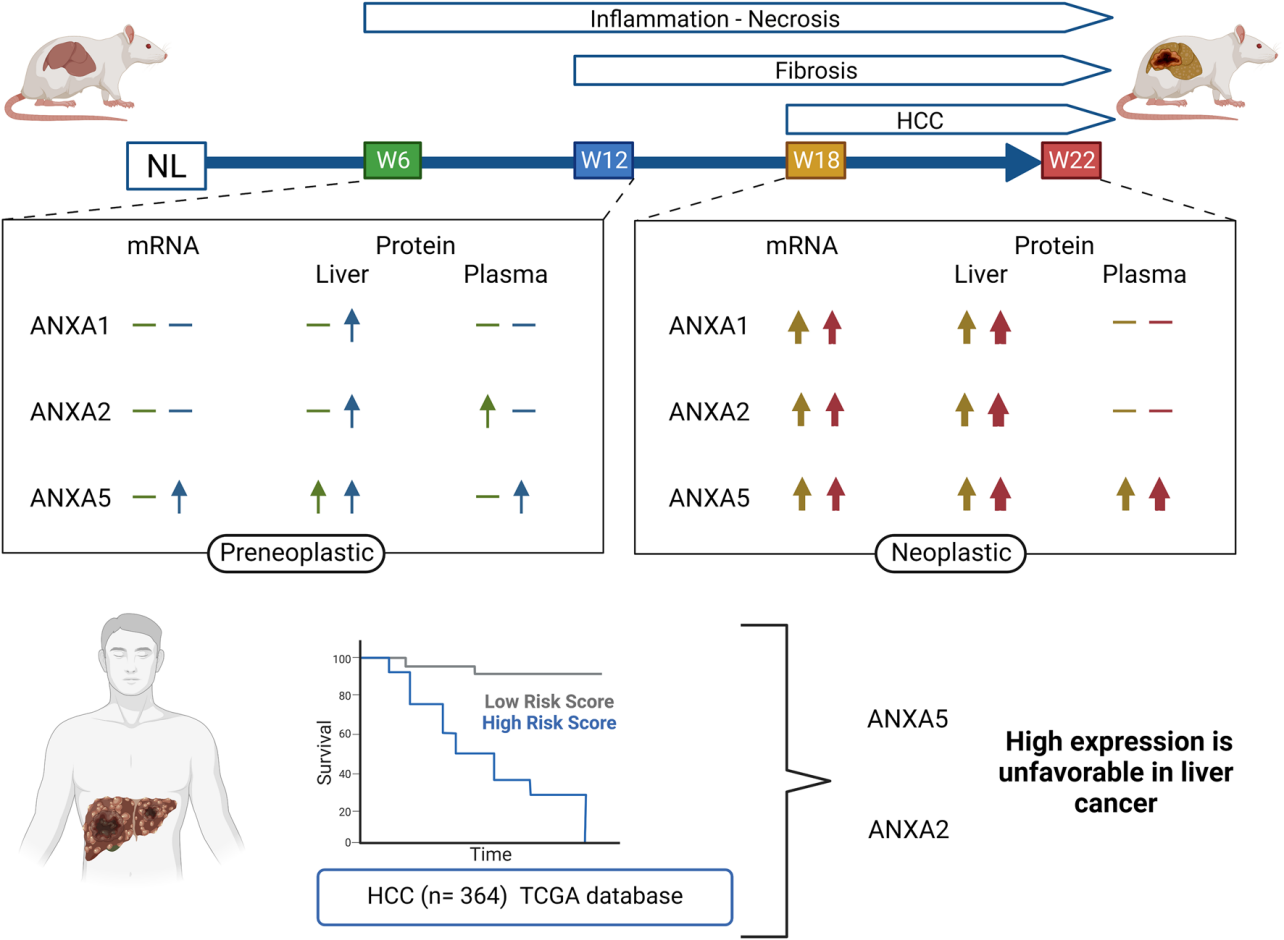
Graphic summary. The summary scheme shows or represents the elevation of ANXA1, ANXA2, and ANXA5 during experimental hepatocarcinogenesis and its association with survival. *NL* normal liver, *W* week. Created with BioRender.com.

Annexin gene expression and protein analysis shown here suggest that ANXA5 and ANXA2 are potential early biomarkers of HCC (Fig. 7). In addition, our work opens avenues for future research for the development of a possible annexin plasma panel that allows the detection of early HCC, as well as the evaluation of these proteins in clinical trials, to determine their sensitivity with concerning AFP.

## Materials and methods

### Animals and ethical approval

For this experiment, fifty-one (51) F344 male rats, weighing 180–200 g, were obtained from the Unit of Production and Experimentation of Laboratory Animals of the Center for Research and Advanced Studies of the National Polytechnic Institute (UPEAL-CINVESTAV-IPN; Mexico City, Mexico). All experiments were performed under the Institutional Animal Care and Use Committee Guidelines and according to protocol No. 0283-18, approved by the Committee for the Care and Use of Laboratory Animals (CICUAL) of CINVESTAV-IPN. Furthermore, the study conformed to the ARRIVE guidelines (Animal Research: Reporting of In Vivo Experiments). Rats were maintained in light/dark cycles of 12 h and controlled temperature in ventilated cages kept in bio-bubbles and protected by a double HEPA filtration system. Animals received a standard diet (PicoLab Rodent Diet 20 5053) and filtered water ad libitum, which was subjected to chlorination and ultrafiltration with a 9 ppm system. The acclimatization period for all animals was 7 days.

### Induction of experimental hepatocarcinogenesis

The hepatocarcinogenesis protocol (Supplementary Fig. S1a) was previously described by Schiffer and coworkers^7^. The rats were divided into five groups at random. Rats were weekly subjected to intraperitoneal injections of DEN (50 mg/kg, Sigma–Aldrich, Toluca, Mexico) for 6 weeks (W6) (n = 9), 12 weeks (W12) (n = 9) for these groups, euthanasia was 2 weeks after the last DEN administration. According to the euthanasia time, the rats of the latter groups were identified as either week 18 (W18) (n = 12) and week 22 (W22) (n = 12) (2 and 6 weeks after the last DEN injection, respectively). The control group (named normal liver; NL) (n = 9) was subjected to 16 injections of the vehicle (0.9% saline) and euthanized six weeks after the last injection.

### Processing of biological material

Animals were euthanized by exsanguination under ketamine (100 mg/kg body weight) and xylazine (8 mg/kg body weight) anesthesia according to the experimental design as illustrated in Supplementary Fig. S1a. Blood was obtained by cardiac puncture, mixed with heparin sodium [1000 UI/mL], (Laboratorios PISA, S.A. DE C.V), and centrifuged for 15 min at 1166 g force. Plasma was separated from the cell package and stored in 500 µL aliquots at − 70 °C. Livers were excised, washed with cold PBS, weighed, photographed, and portioned for different purposes. Some liver fractions were frozen in 2-methyl butane with liquid nitrogen and stored at − 70 °C; other pieces were fixed in 10% formalin, embedded in paraffin, and sectioned for further histological analyses.

### Histological and immunofluorescence analyses

Embedded tissues were cut into 5 μm thick and placed in electrocharged slides; then, histological sections were deparaffinized and gradually rehydrated. For histological examination, samples were stained using the standard H&E method. For IF, after sample rehydration, antigen was retrieved according to the Rodent Decloaker kit (BioCare Medical. CA, USA). Then, they were blocked with 1% BSA for 2 h and incubated overnight at 4 °C with the following primary antibodies: Annexin A1 (ANXA1, 1:50; NBP1-90162; RRID: AB_11018791), Annexin A2 (ANXA2, 1:50; NBP1-31310; RRID: AB_2242775), and Annexin A5 (ANXA5, 1:50; NBP2-38248); all antibodies were from NOVUS Biologicals. CO, USA. Then, tissues were incubated for 1 h at room temperature in the dark with an Alexa Fluor 488 anti-rabbit antibody (1:300, ab150077; RRID: AB_2630356, Abcam, MA, USA). Next, nuclear DNA was stained with DAPI (1:1000; 62.247; Thermo Scientific, IL, USA) for 5 min at room temperature. Finally, tissues were fixed with Fluoroshield mounting medium (Thermo Scientific, IL, USA), and IF images were captured with ZEISS Axio-A1 Microscopy (Carl-Zeiss, Oberkochen, Germany).

### Liver protein extraction

Total proteins were extracted from liver tissue slices cut with a cryostat at − 16 °C; thick sections of 15–20 μm were collected in tubes until reaching 50 mg. Later this tissue was subjected to homogenization with 1 mL of lysis buffer containing Tris–HCl [20 mM], NaCl [120 mM], EDTA [2 mM], Glycerol [10%], Nonident-P40 [1%], and protease inhibitors cocktail (complete and PhoSTOP as indicated by Roche). Then, samples were kept in an orbital shaker at 4 °C for 30 min and centrifuged at 13,709 *g* force for 20 min. Finally, the supernatant was set aside in sterilized tubes, and 20 μL aliquots were stored at − 70 °C for further analyses.

Cytosolic and nuclear protein fractions were isolated from 100 mg of frozen tissues homogenized in 1 mL of lysis buffer A containing HEPES [10 mM, pH 7.9], KCl [10 mM], EDTA [0.1 mM], EGTA [0.1 mM], Nonident-P40 [0.15%], and protease inhibitors. Samples were incubated on ice for 15 min, centrifuged at 16,089 *g* force for 15 s, and the supernatant was separated as the cytosolic protein fraction and stored at − 70 °C. The nuclei contained in the pellet were washed three times with buffer A and homogenized in 300 µL of lysis buffer B containing HEPES [20 mM, pH 7.9], NaCl [0.4 mM], EDTA [ 1 mM], EGTA [1 mM], Nonident-P40 [5%], and protease inhibitors. Samples were kept in an orbital shaker at 4 °C for 15 min and centrifuged at 16,089 *g* force for 30 min. Then, the supernatant was separated as the nuclear protein fraction and stored at − 70 °C for further analyses.

### Western blot (WB) analysis

The total protein content was determined by Bicinchoninic Acid (BCA) assay (B9643-1L; Sigma–Aldrich, Toluca, Mexico). Thirty micrograms of protein per sample were separated by SDS-PAGE and transferred to a PVDF membrane (Millipore, MA, USA). Membranes were blocked for 1 h at room temperature with 5% skimmed milk and then incubated overnight at 4 °C with primary antibodies against ANXA1 (1:300), ANXA2 (1:500), ANXA5 (1:500), ANXA10 (1:500; NBP1-90,156; RRID: AB_11004664), and glutathione S-transferases P1, (GSTP1, 1:10,000; NBP2-16,756), from Novus Biologicals; CO, USA; ANXA8 (1:500; ab111693; RRID: AB_10858116), glyceraldehyde-3-phosphate dehydrogenase (GADPH, 1:10,000; ab181602; RRID: AB_2630358), and Lamin β1 (1:10,000; Ab16048; RRID: AB_443298), from Abcam; MA, USA; and prostaglandin reductase 1, (Ptgr1, 1:2600; GTX118527; RRID: AB_10618960) from GeneTex; CA, USA. After washing, membranes were incubated for 1 h with an anti-rabbit peroxidase-conjugated secondary antibody (1:10,000; NB7160; RRID: AB_524669; Novus Biologicals; CO, USA); then, a chemiluminescence reaction was performed by adding the Immobilon Western Chemiluminescent HRP Substrate (WBKLS0500; Millipore. MA, USA). Finally, images were captured with the Uvitec MINI HD6 photo-documentation system (Cambridge, LDN, UK). Relative expression was calculated using ImageJ Software version 1.41 (National Institutes of Health, USA).

### Enzyme-linked immunosorbent assay (ELISA)

Different annexin proteins were quantified by ELISA in both liver tissue and plasma according to the supplier's recommendations. For ANXA1, ANXA2, and ANXA5, the rat ELISA Kit OKEH03091, Lot # KD2382; OKCD02307, Lot # KD5003; and OKEH03075, Lot # KD2381; respectively, from Aviva Systems Biology (San Diego, CA, USA), were used.

### Quantitative RT-PCR analysis

Total RNA was extracted from 20 mg of liver tissue slices cut with a cryostat at − 16 °C that were immediately protected in the RNeasy Mini Kit (Qiagen, 74106; NW; Germany) as indicated by the manufacturer. Concentration and purity were determined spectrophotometrically using the spectrophotometer NanoDrop ND-1000 (Thermo Fisher Scientific Inc; MA, USA), and the rRNA integrity was determined by agarose gel electrophoresis. The cDNA reactions were prepared from 750 ng of total RNA using the High-Capacity Reverse cDNA transcription kit (4,368,814; Applied Biosystems; MA, USA) and used for RT-qPCR.

The RT-qPCR analysis was carried out using TaqMan gene expression assays in the QuantStudio 7 Flex Real-time PCR system (Applied Biosystem, MA, USA). (Supplementary Table S1). Fluorochrome FAM (borderline-exon-exon) labeled probes were obtained for rat *Anxa1* (Rn00563742_m1), rat *Anxa2* (Rn00571516_m1), rat *Anxa5* (Rn00565571_m1) rat *Anxa8* (Rn01756160_m1), rat *Anxa10* (Rn01402905_m1) and *18S* rRNA (Rn0392899_g1), and the reaction was performed following the supplier’s recommendations. Relative gene expression values were calculated according to the (ΔΔCt) method^45^, where the *18s* rRNA was used as internal control, and the NL group was used as a sample reference.

### Kaplan–Meier survival analysis

We investigated the relationship between gene expression levels of *ANXA1, ANXA2*, *ANXA5*, *PTGR1*, and *GSTP1* and overall survival in patients diagnosed with HCC. For our analysis, we used a sample group obtained from the Liver Hepatocellular Carcinoma TCGA PanCancer data. (https://www.cbioportal.org/study/summary?id=lihc_tcga_pan_can_atlas_2018).

Initially, the sample comprised 372 patients. However, we excluded three patients diagnosed with the fibrolamellar carcinoma subtype and five patients who lacked the copy-number alterations data. As a result, our final sample group consisted of 364 patients, representing 98.6% of the HCC database. We analyzed the data using the cBioPortal platform (https://www.cbioportal.org/) and classified the cases into high or low-expression groups based on the mean expression level of each gene. Finally, we used the log-rank test (*p* < 0.05) to investigate the correlation between gene expression and overall survival.

### Statistical analyses

Statistical analyzes were performed using GraphPad Prism version 8.0 Software (GraphPad Software, San Diego, CA, USA). For multiple comparisons, statistical differences were obtained by one-way ANOVA and Tukey's post hoc test. Statistical significance was considered when *p* < 0.05.

## Supplementary Information

Supplementary Information.

## Data Availability

All datasets used and/or analyzed during the current study are available upon reasonable request to the corresponding author.
