# Supplementary material for: Annexins A2 and A5 are potential early biomarkers of hepatocarcinogenesis
Source: Sci Rep. 2023 Apr 28;13:6948. doi: 10.1038/s41598-023-34117-8 (PMC10147597; doi:10.1038/s41598-023-34117-8)

Supplementary Information

Annexins A2 and A5 are potential early biomarkers of hepatocarcinogenesis

Ema Elvira Herrera-López, Dafne Guerrero-Escalera, Isaac Aguirre-Maldonado, Arely López-Hernández, Hilda Montero, María Angélica Gutiérrez-Nava, Luis del Pozo-Yauner, Jaime Arellanes-Robledo, Javier Camacho, Julio Isael Pérez-Carreón*

* Corresponding author: Julio Isael Pérez-Carreón. Instituto Nacional de Medicina Genómica. Periférico Sur No. 4809, Col. Arenal Tepepan, Alcaldía Tlalpan. México, D.F. C.P. 14610. Tel. +52 (55) 5350-1900 ext. 1170, Fax 5350-1999. E-mail: jiperez@inmegen.gob.mx

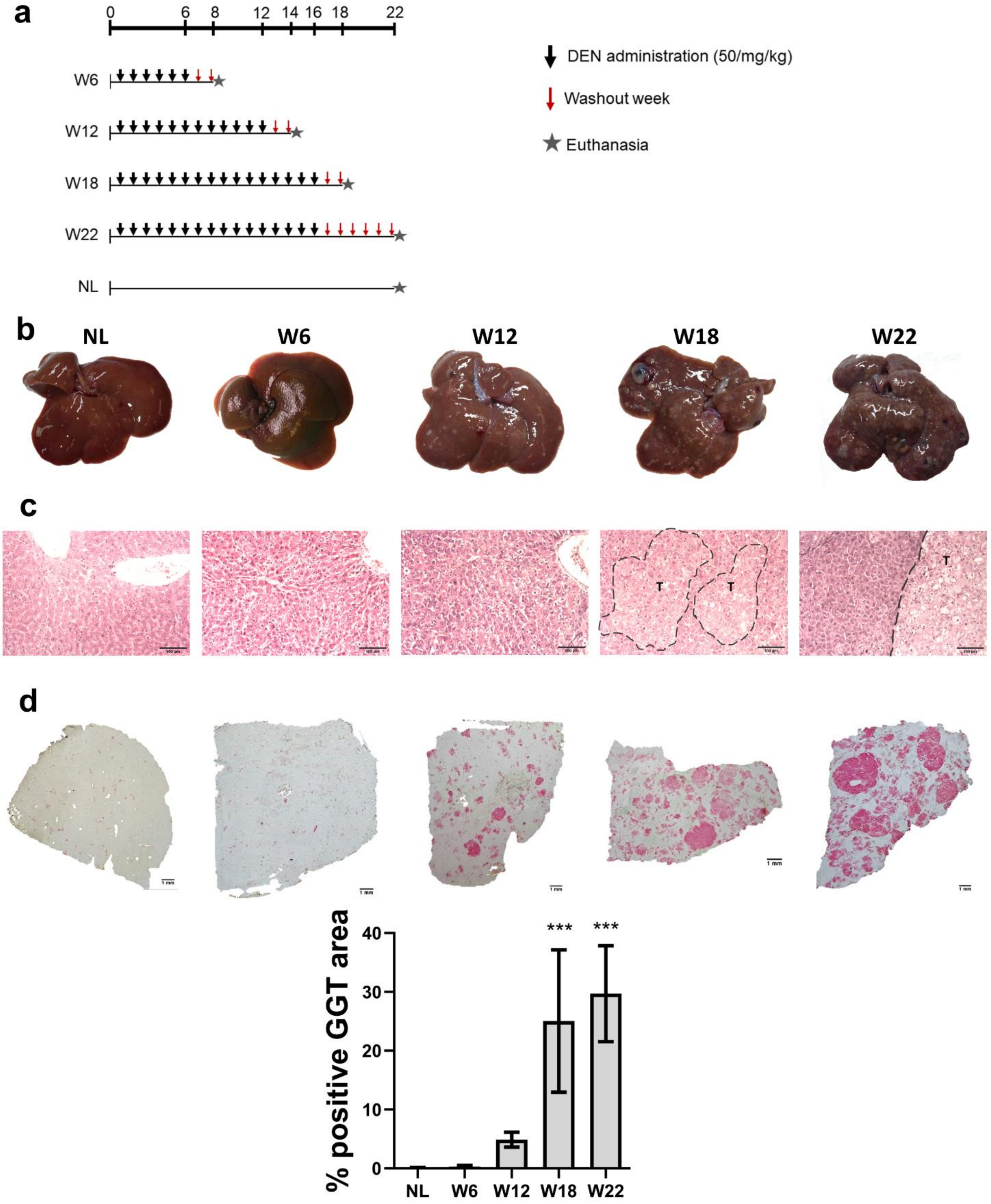

a
0
6
8
12
14
16
18
22
W6
W12
W18
W22
NL
DEN administration (50/mg/kg)
Washout week
Euthanasia
b
NL
W6
W12
W18
W22
c
T
T
T
d
1 mm
% positive GGT area
40
30
20
10
0
***
***
NL
W6
W12
W18
W22

Figure S1. **Experimental design and sequential HCC progression in the rat model.** (a) The hepatocarcinogenesis protocol shows that DEN (50 mg/kg) was intraperitoneally administrated once a week until 16 weeks, as indicated by arrows. Rats were subjected to euthanasia after two weeks of the last DEN administration, i.e., at 8, 14, and 18, respectively, except for 22 weeks group, which was euthanized six weeks after the last DEN dose. NL group was subjected to 16 injections of the vehicle (0.9% saline) and was euthanized six weeks after the last injection. (b) Representative images of livers. (c) H&E- stained sections. Magnification: 200X. Scale bar = 100 μm. (d) Histological sections show GGT-positive nodules and tumors, Scale bar = 1 mm. DEN, diethylnitrosamine; NL, normal liver; W, week; T, tumor.

Supplementary Table 1. TaqMan Gene Expression Assay Information

Gene Name	Gene Symbol:	Assay ID	5’ reporter dye	3' nonfluorescent quencher	RefSeq	Exon Boundary	Assay Location	Amplicon Length
annexin A1	*Anxa1*	Rn00563742_m1	FAM	MGB	NM_012904.2	8 - 9	669	81
annexin A2	*Anxa2*	Rn00571516_m1	FAM	MGB	NM_019905.1	7 - 8	571	78
annexin A5	*Anxa5*	Rn00565571_m1	FAM	MGB	NM_013132.1	4 - 5	252	115
annexin A8	*Anxa8*	Rn01756160_m1	FAM	MGB	NM_001031654.1	3 - 4	228	66
annexin A10	*Anxa10*	Rn01402905_m1	FAM	MGB	NM_001109110.1		647	67
18S ribosomal RNA	*Rn18s*	Rn03928990_g1	FAM	MGB	NR_046237.1	1	152	61

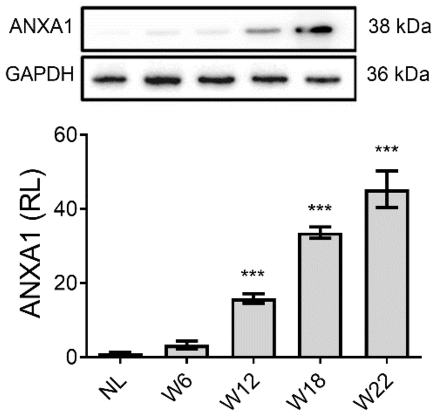

Original blots of Figure 2

General information

Annexin A1. ANXA1 [1:300] NBP1-90162; RRID: AB_11018791
1-second exposure time
Theoretical MW: 38 KDa
Supplier-reported MW: 35-55 KDa
Estimation MW:36.8 KDa

GAPDH. [1:10000]; ab181602; RRID: AB_2630358
2-second exposure time
Theoretical MW: 36 KDa
Supplier-reported MW: 25-37 KDa
Estimation MW: 36.9 KDa

Original images captured with the Uvitec MINI HD6 photo documentation system.

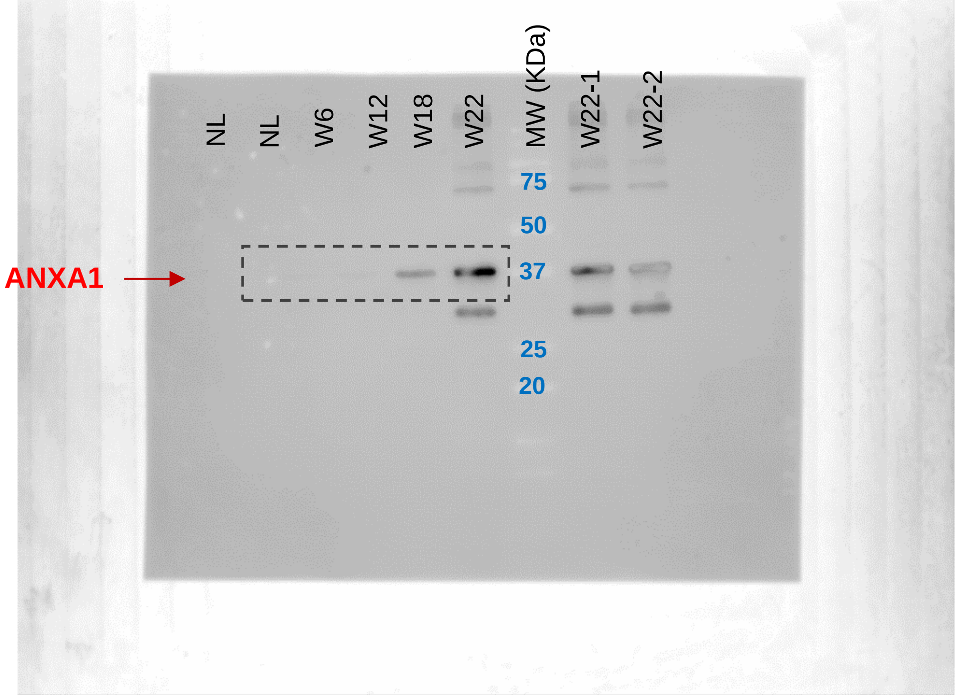

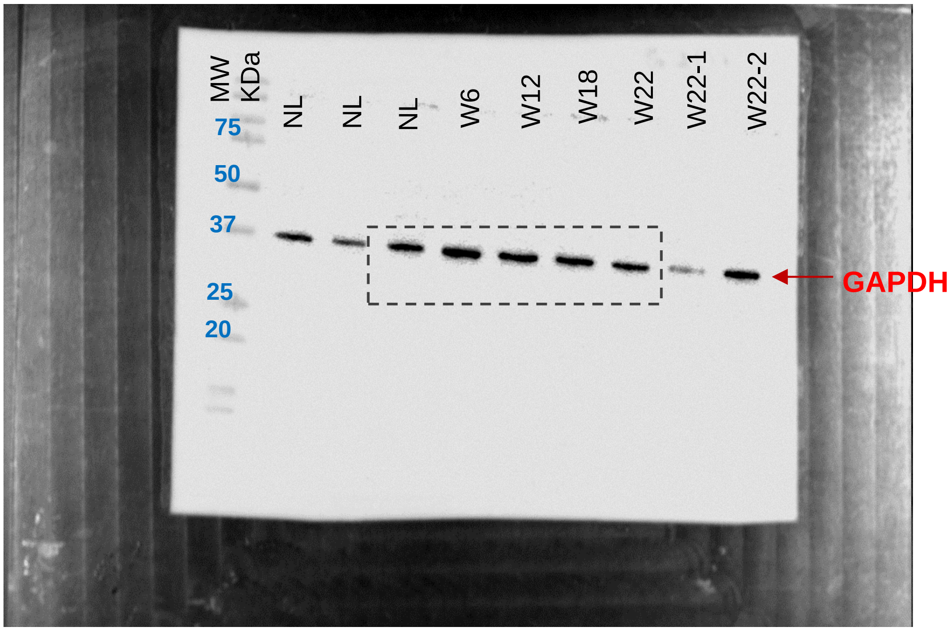

Original blots of Figure 2

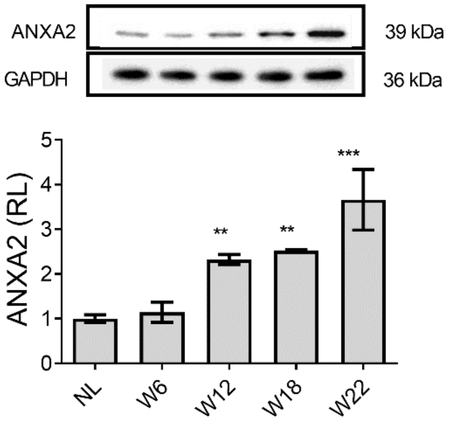

General information

Annexin A2. ANXA2 [1:500] NBP1-31310; RRID: AB_2242775
1-second exposure time
Theoretical molecular weight (MW): 39 KDa
Supplier-reported MW: 34-43 KDa
Estimation MW: 40.9 KDa

GAPDH, [1:10000]; ab181602; RRID: AB_2630358
5-second exposure time
Theoretical MW: 36 KDa
Supplier-reported MW: 25-37 KDa
Estimation MW: 36.9 KDa

Original images captured with the Uvitec MINI HD6 photo documentation system.

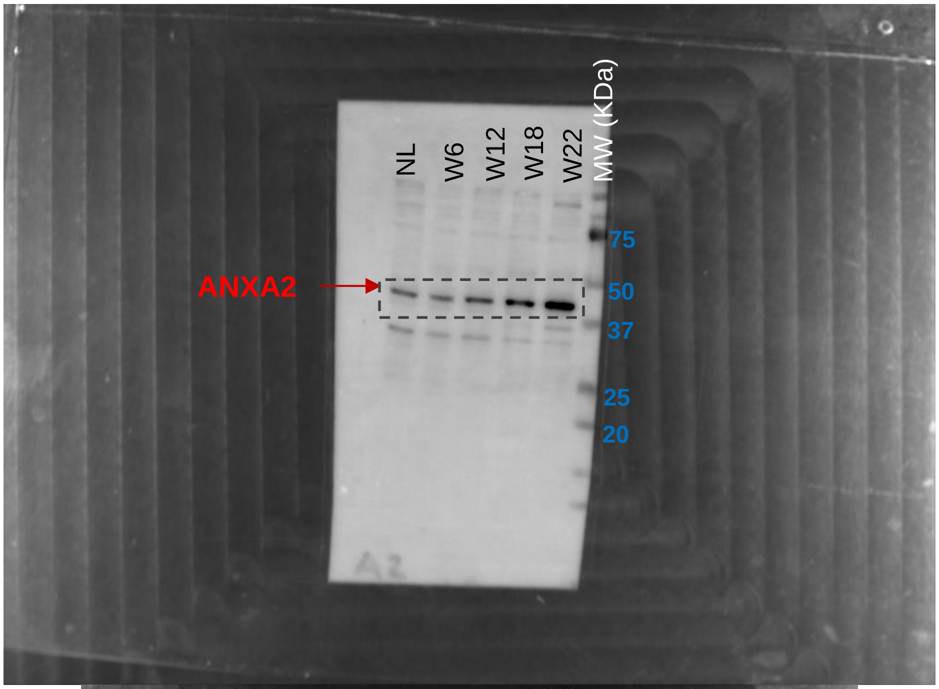

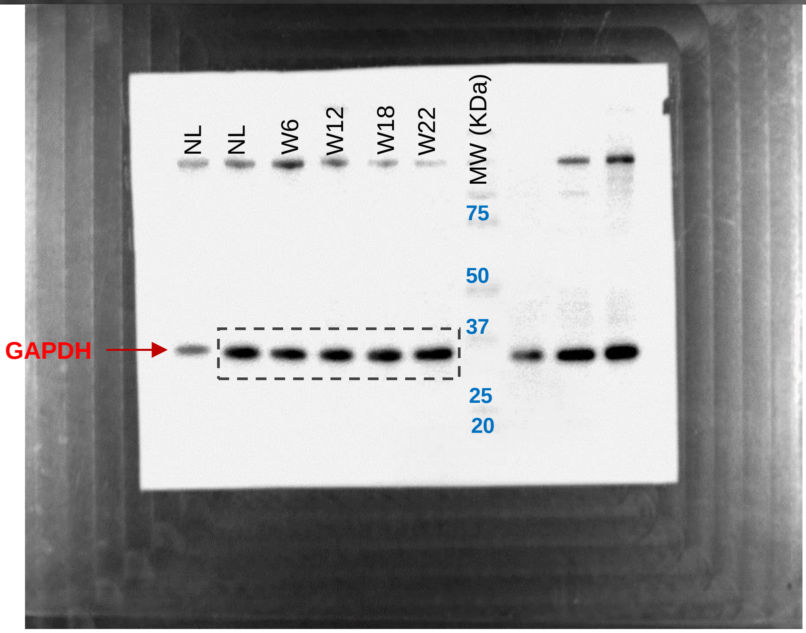

Original blots of Figure 2

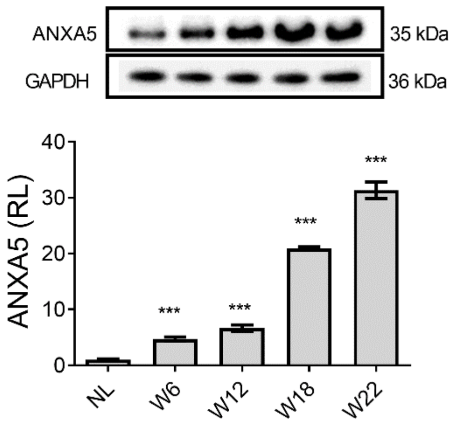

General information

Annexin A5. ANXA5 [1:500] NBP2-38248
6-second exposure time
Theoretical MW: 35 KDa
Supplier-reported MW: 28-36 KDa
Estimation MW: 33.3 KDa

GAPDH, [1:10000]; ab181602; RRID: AB_2630358
5-second exposure time
Theoretical MW: 36 KDa
Supplier-reported MW: 25-37 KDa
Estimation MW: 36.9 KDa

Original images captured with the Uvitec MINI HD6 photo documentation system.

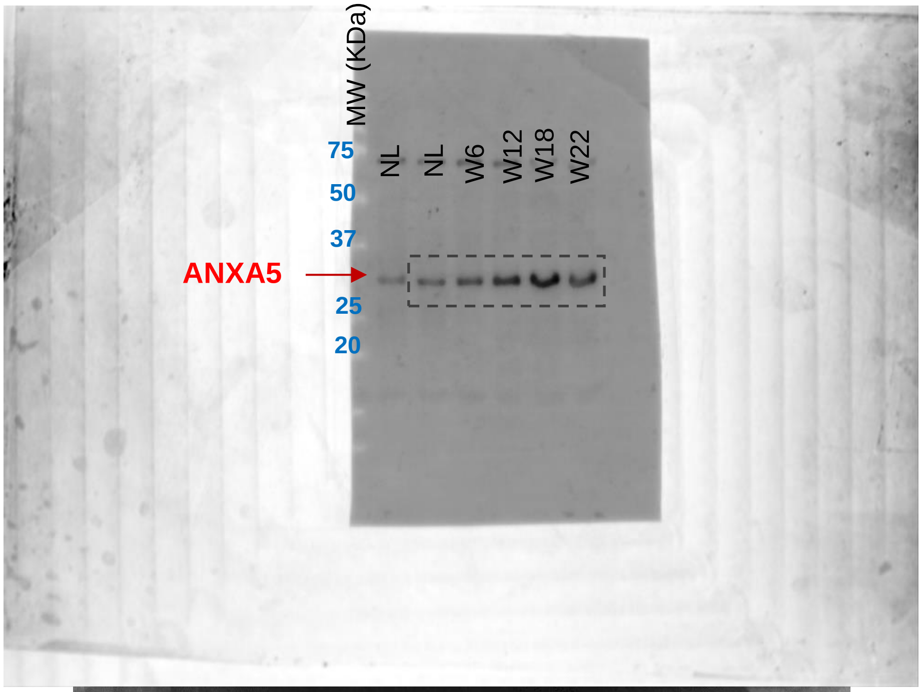

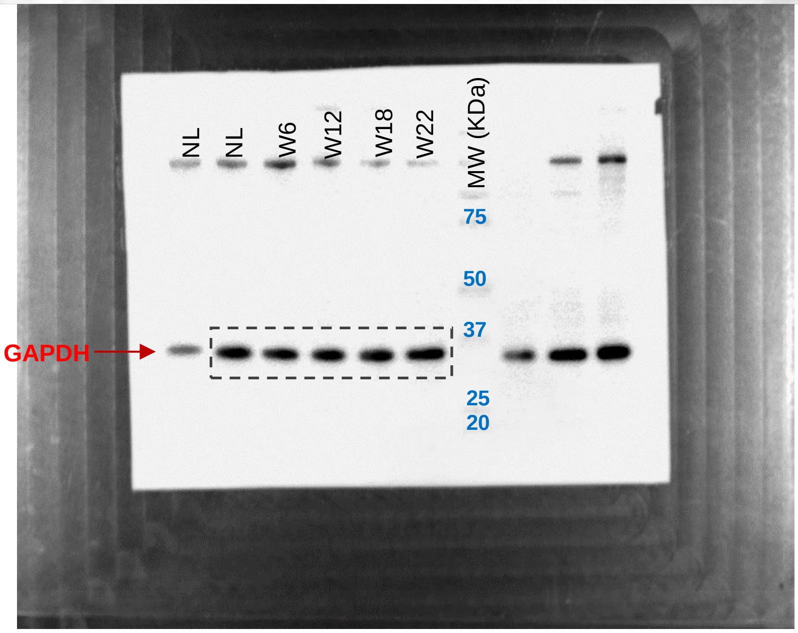

Original blots of Figure 2

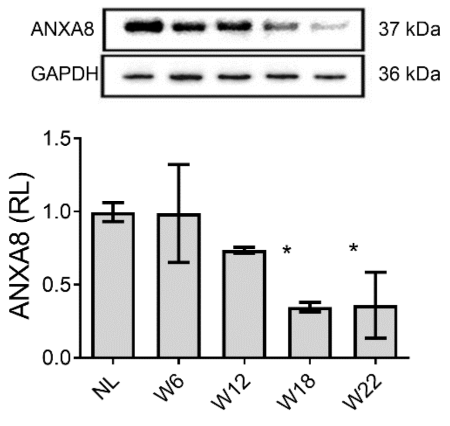

General information

Annexin A8. ANXA8 [1:500]; ab111693; RRID: AB_10858116
9-second exposure time
Theoretical MW: 37 KDa
Supplier-reported MW: 34-43 KDa
Estimation MW: 42.3 KDa

GAPDH, [1:10000]; ab181602; RRID: AB_2630358
9-second exposure time
Theoretical MW: 36 KDa
Supplier-reported MW: 25-37 KDa
Estimation MW: 36.9 KDa

Original images captured with the Uvitec MINI HD6 photo documentation system.

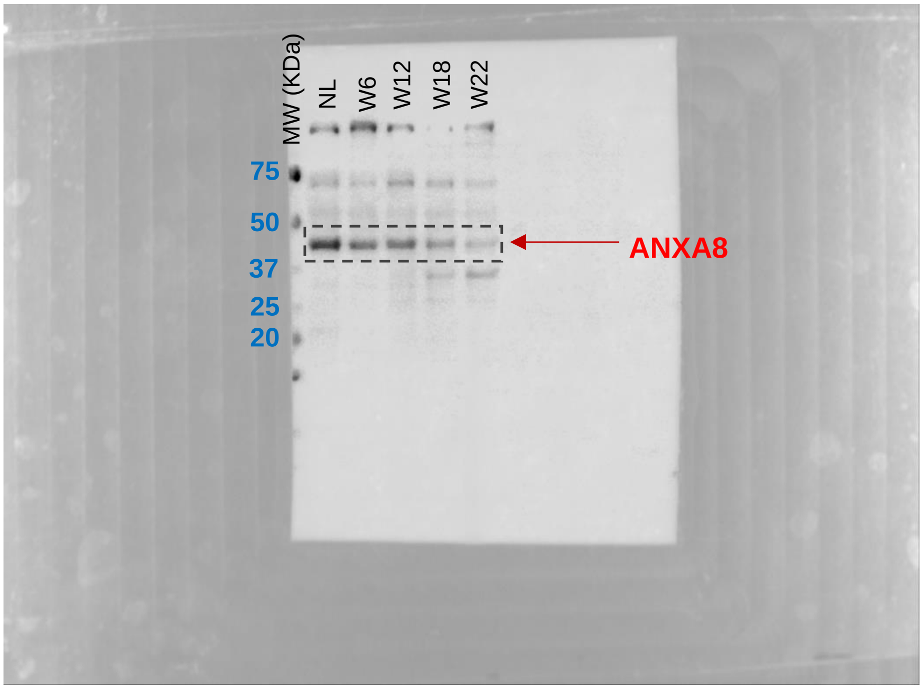

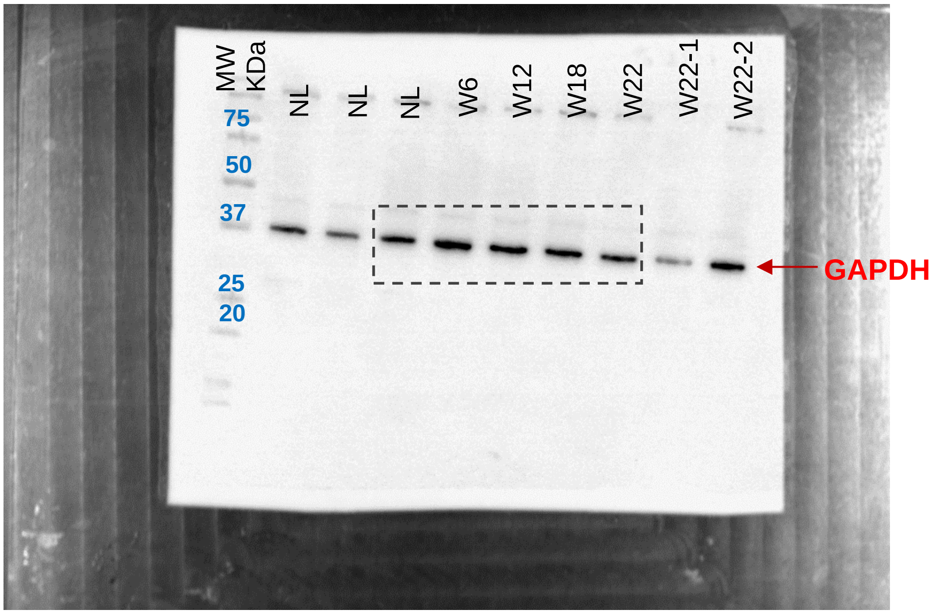

Original blots of Figure 2

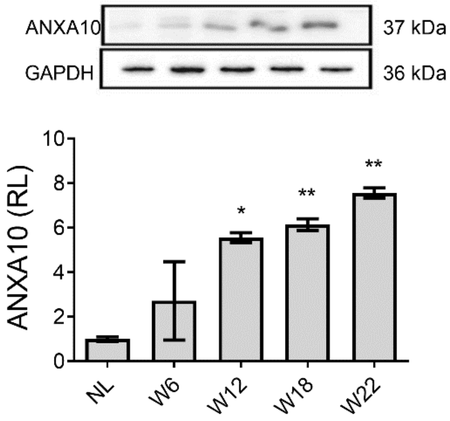

General information

Annexin A10. ANXA10 [1:500]; NBP1-90156; RRID: AB_11004664
12-second exposure time
Theoretical MW: 37 KDa
Supplier-reported MW: 25-35 KDa
Estimation MW:31.9 KDa

GAPDH, [1:10000]; ab181602; RRID: AB_2630358
9-second exposure time
Theoretical MW: 36 KDa
Supplier-reported MW: 25-37 KDa
Estimation MW: 36.9 KDa

Original images captured with the Uvitec MINI HD6 photo documentation system.

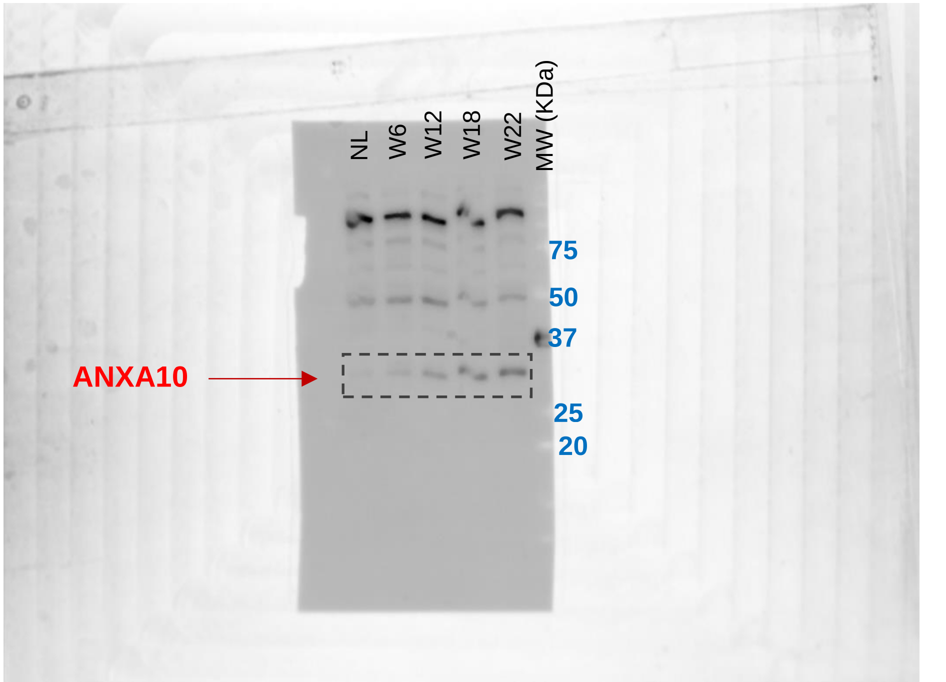

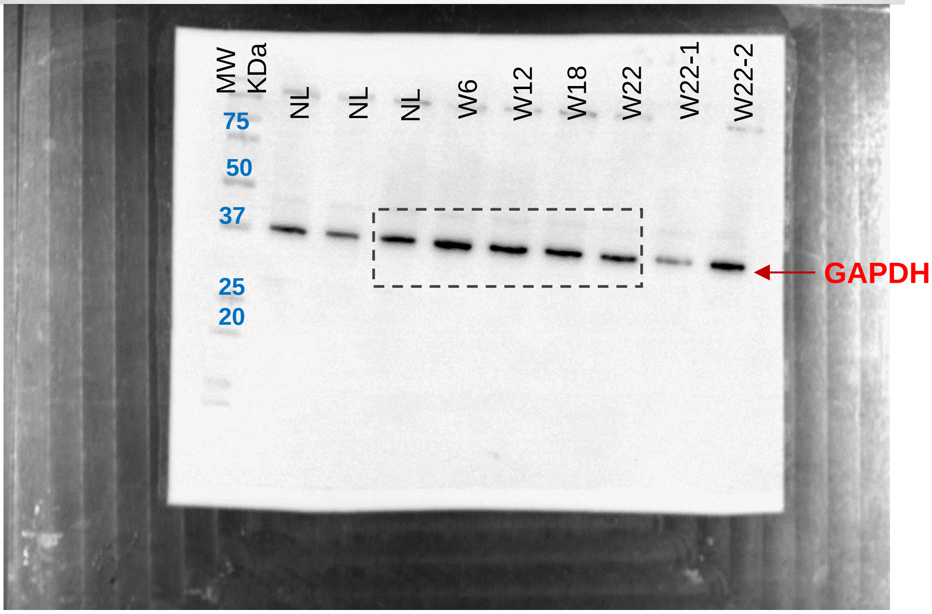

Original blots of Figure 2

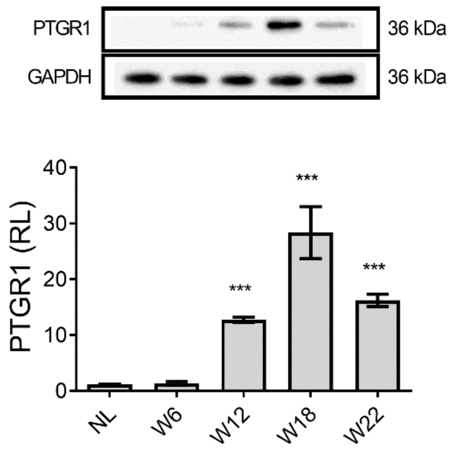

General information

PTGR1. (1:2600); GTX118527; RRID: AB_10618960
6-second exposure time
Theoretical MW: 36 KDa
Supplier-reported MW: 34-43 KDa
Estimation MW: 33.8 KDa

GAPDH. [1:10000]; ab181602; RRID: AB_2630358
5-second exposure time
Theoretical MW: 36 KDa
Supplier-reported MW: 25-37 KDa
Estimation MW: 36.9 KDa

Original images captured with the Uvitec MINI HD6 photo documentation system.

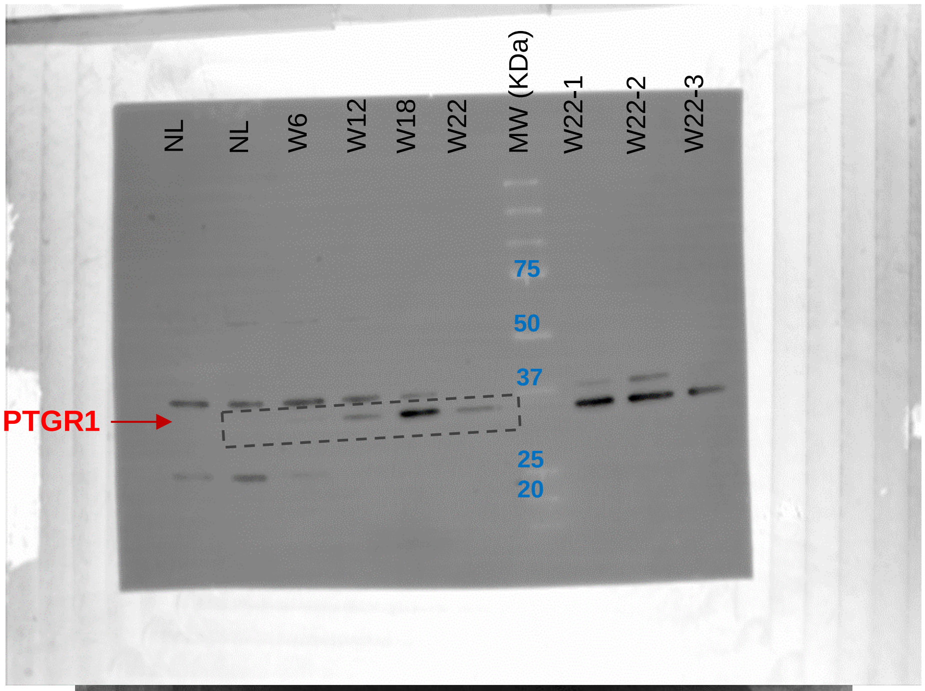

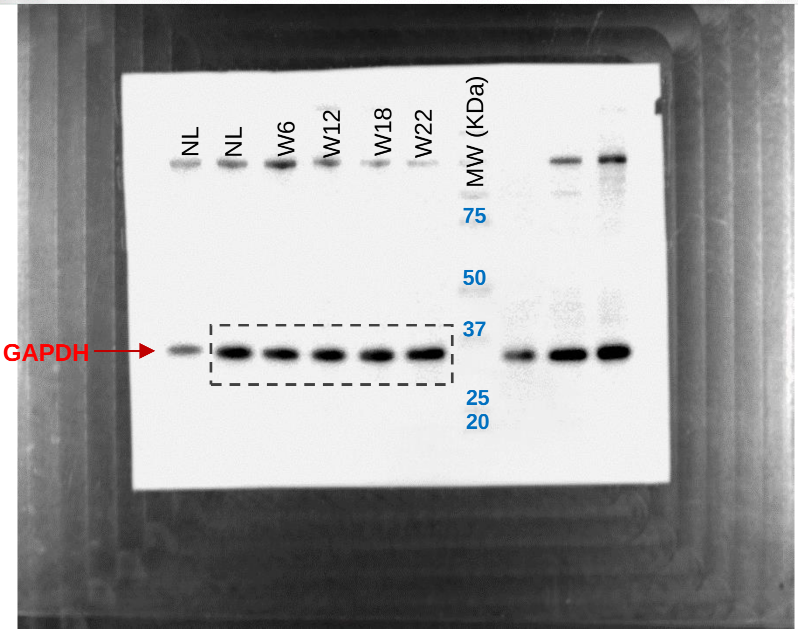

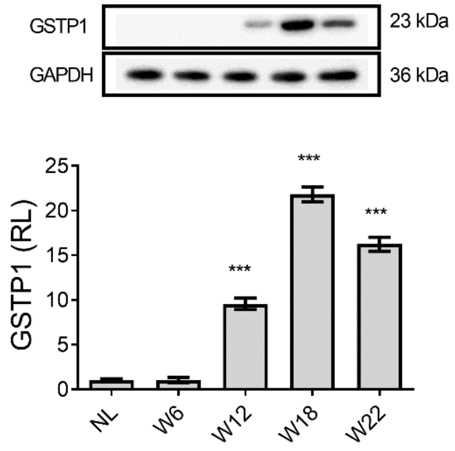

Original blots of Figure 2

General information

GSTP1. [1:10000]; NBP2-16756
5-second exposure time
Theoretical MW: 23 KDa
Supplier-reported MW: 15-25 KDa
Estimation MW: 23.6 KDa

GAPDH, [1:10000]; ab181602; RRID: AB_2630358
5-second exposure time
Theoretical MW: 36 KDa
Supplier-reported MW: 25-37 KDa
Estimation MW: 36.9 KDa

Original images captured with the Uvitec MINI HD6 photo documentation system.

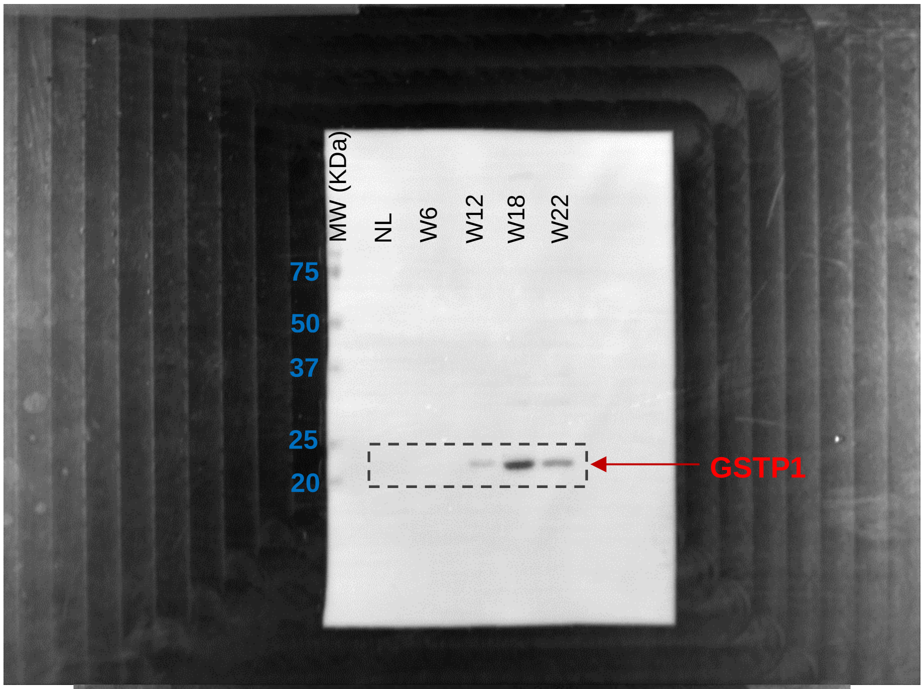

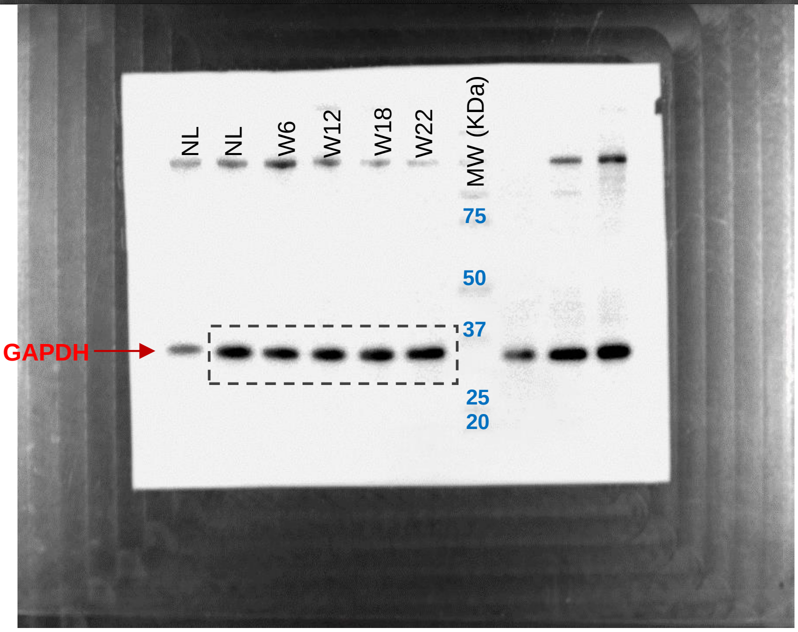

Original blots of Figure 4

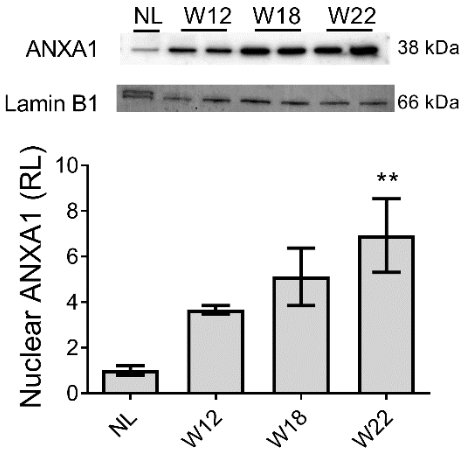

General information

Annexin A1 Nuclear. ANXA1 [1:300] NBP1-90162; RRID: AB_11018791
15-second exposure time
Theoretical MW: 38 KDa
Supplier-reported MW: 35-55 KDa
Estimation MW:37 KDa

Lamin β1. [1:10000]; Ab16048; RRID: AB_443298
5-min exposure time
Theoretical MW: 66 KDa
Supplier-reported MW: 50-75 KDa
Estimation MW: 66.3 KDa

Original images captured with the Uvitec MINI HD6 photo documentation system.

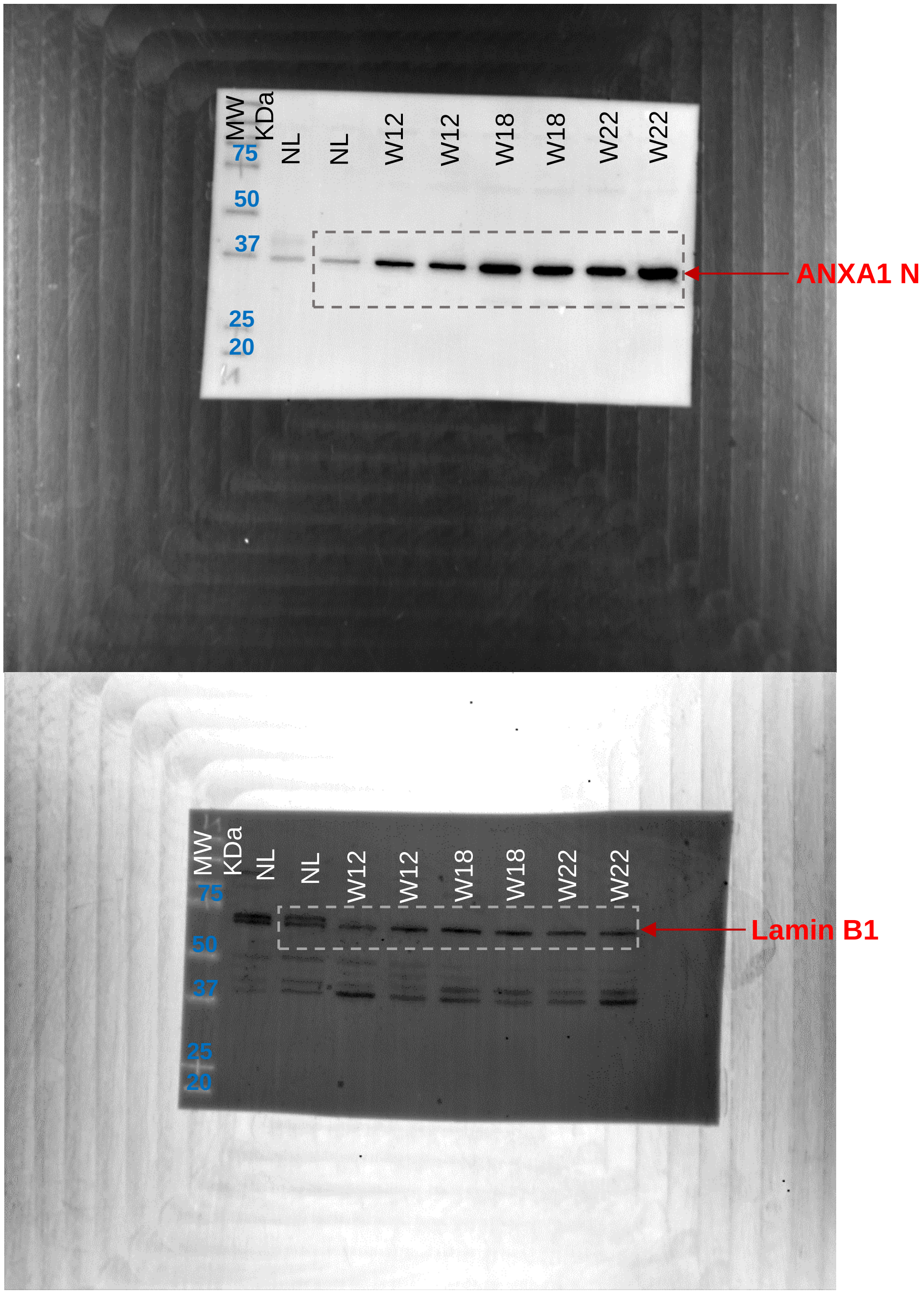

Original blots of Figure 4

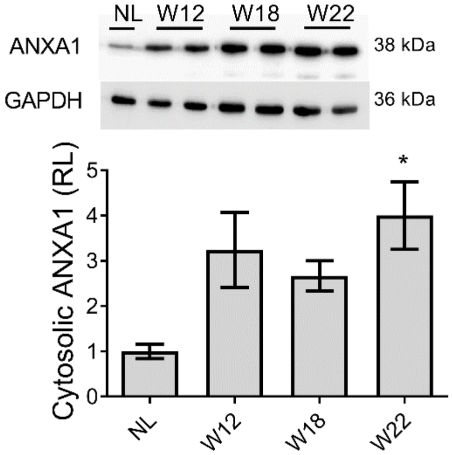

General information

Annexin A1 Cytosolic. ANXA1 [1:300] NBP1-90162; RRID: AB_11018791
10-second exposure time
Theoretical MW: 38 KDa
Supplier-reported MW: 35-55 KDa
Estimation MW:36.8 KDa

GAPDH. [1:10000]; ab181602; RRID: AB_2630358
4-second exposure time
Theoretical MW: 36 KDa
Supplier-reported MW: 25-37 KDa
Estimation MW: 36.6 KDa

Original images captured with the Uvitec MINI HD6 photo documentation system.

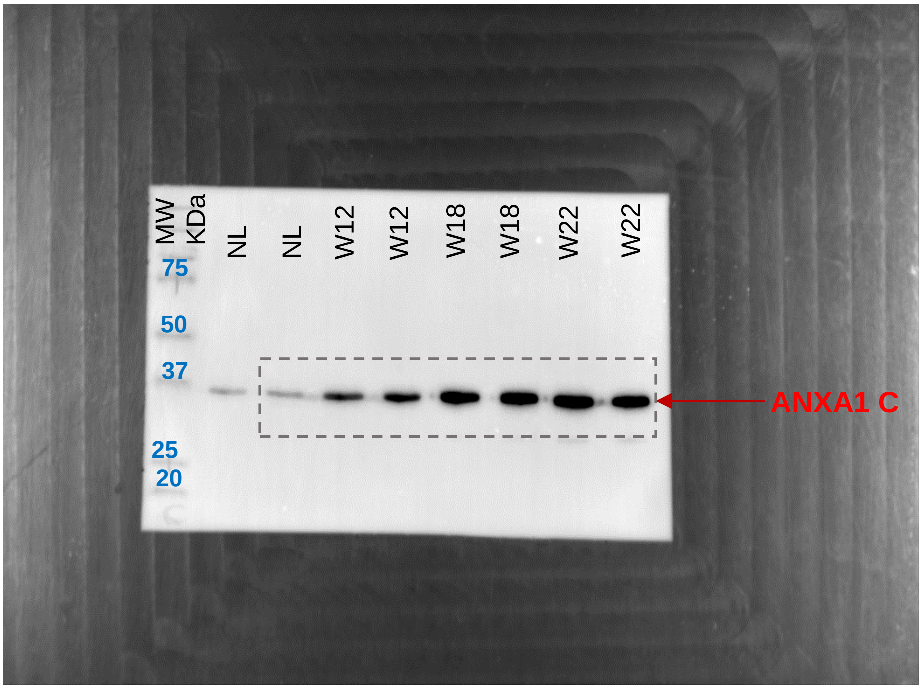

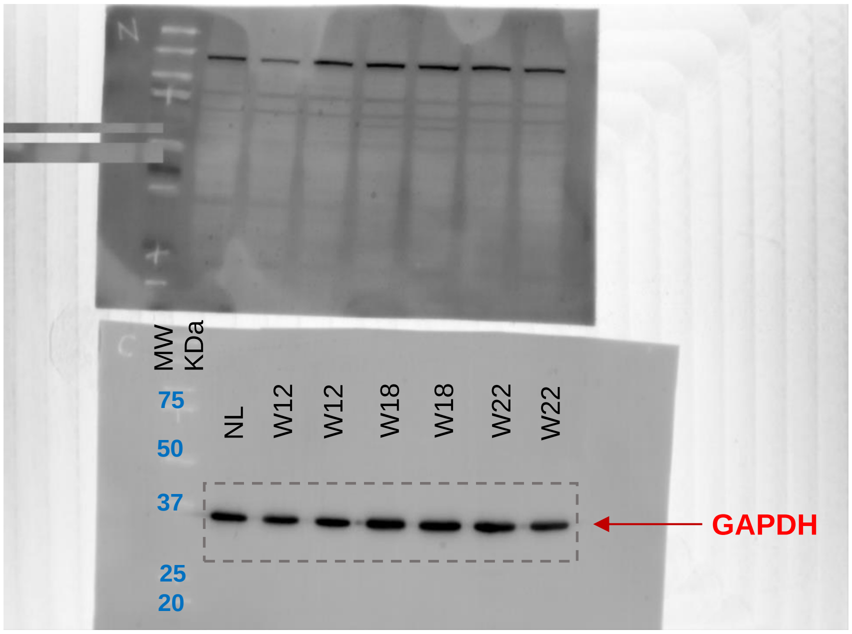

Original blots of Figure 4

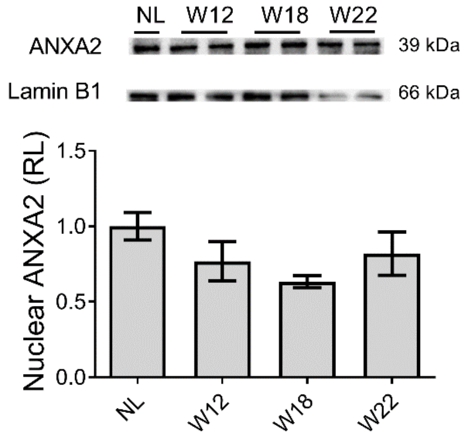

General information

Annexin A2 Nuclear. ANXA1 [1:300] NBP1-90162; RRID: AB_11018791
4-second exposure time
Theoretical MW: 39 KDa
Supplier-reported MW: 35-55 KDa
Estimation MW:36.8 KDa

Lamin β1. [1:10000]; Ab16048; RRID: AB_443298
2-min exposure time
Theoretical MW: 66 KDa
Supplier-reported MW: 50-75 KDa
Estimation MW: 67.4 KDa

Original images captured with the Uvitec MINI HD6 photo documentation system.

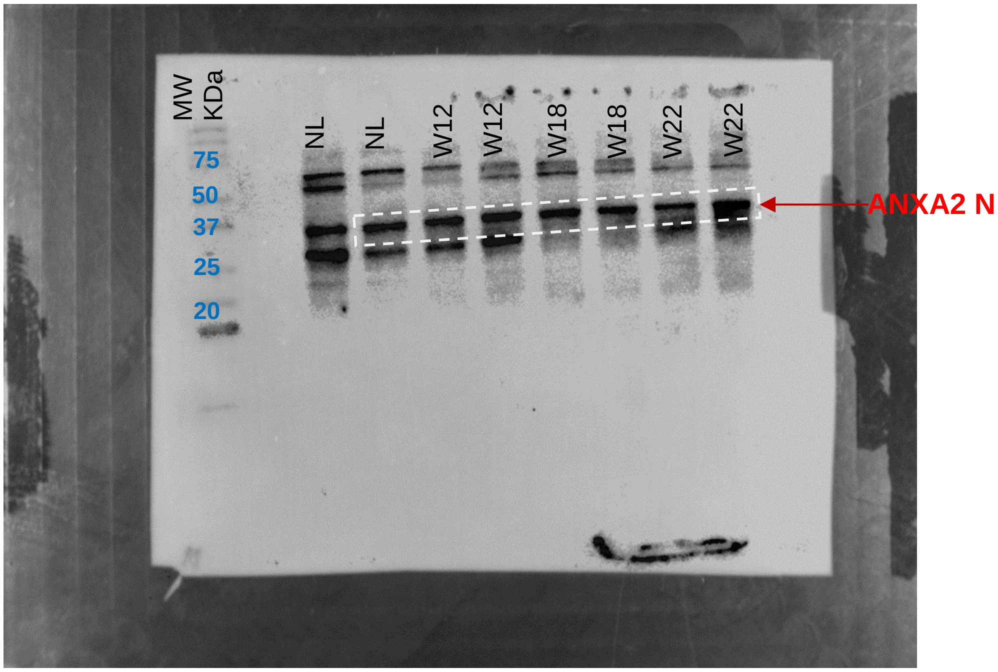

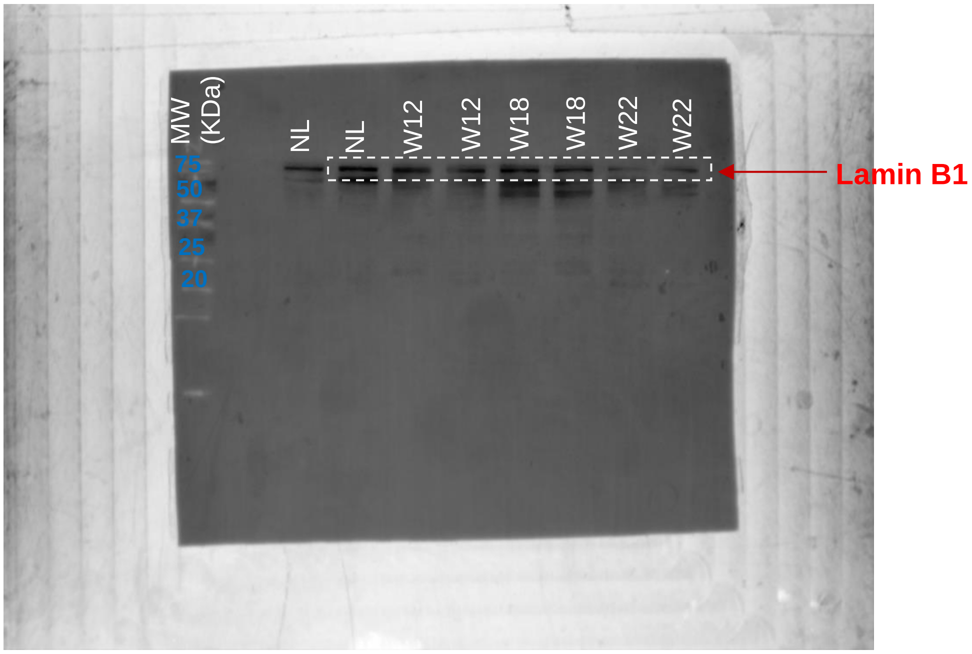

Original blots of Figure 4

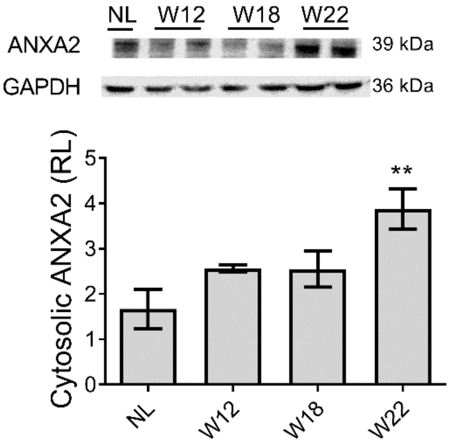

General information

Annexin A2 Cytosolic. ANXA1 [1:300] NBP1-90162; RRID: AB_11018791
10-second exposure time
Theoretical MW: 39 KDa
Supplier-reported MW: 35-55 KDa
Estimation MW:36.2 KDa

GAPDH. [1:10000]; ab181602; RRID: AB_2630358
5-second exposure time
Theoretical MW: 36 KDa
Supplier-reported MW: 25-37 KDa
Estimation MW: 36.8 KDa

Original images captured with the Uvitec MINI HD6 photo documentation system.

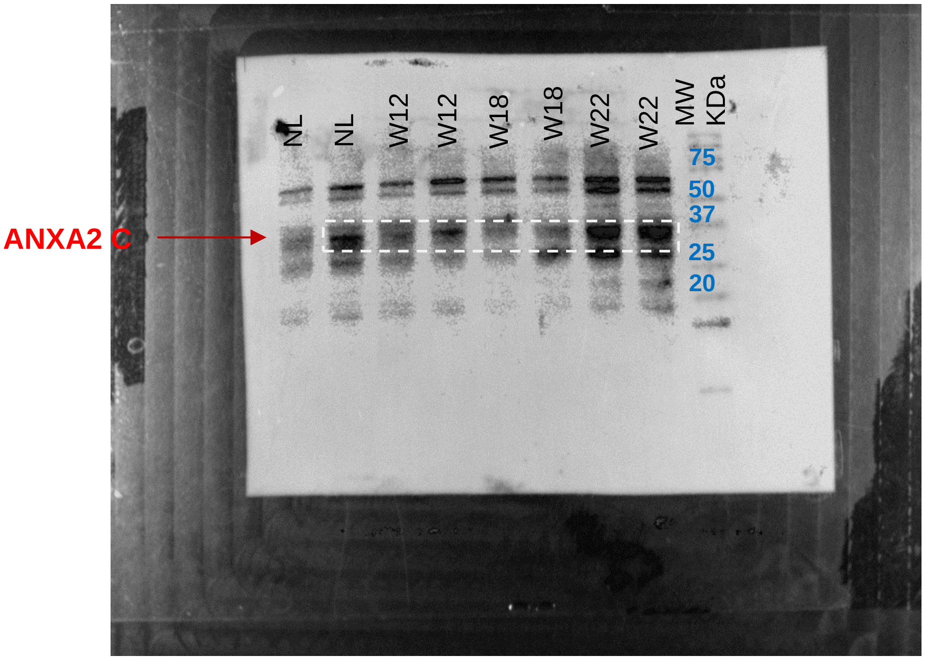

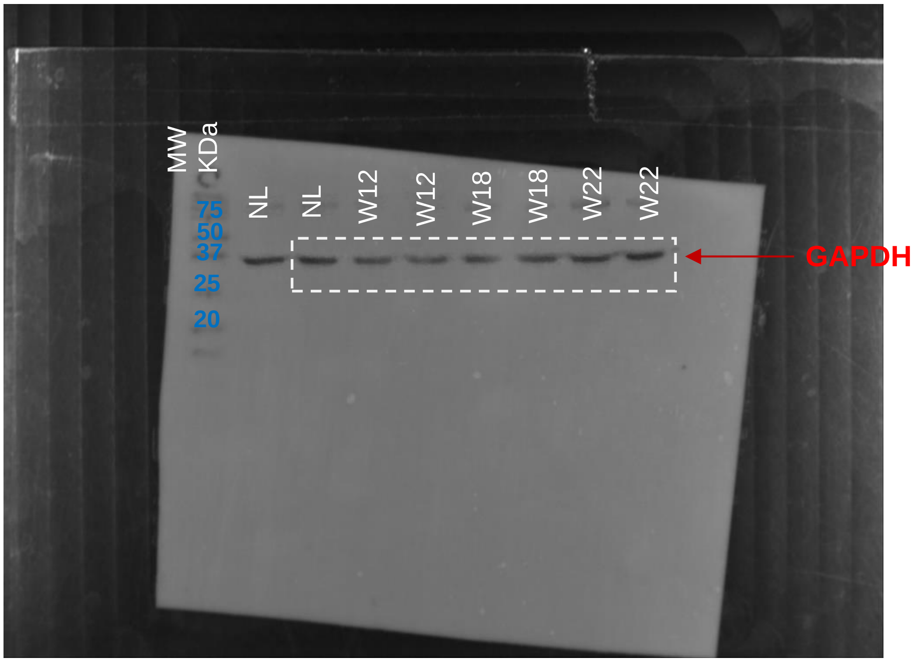

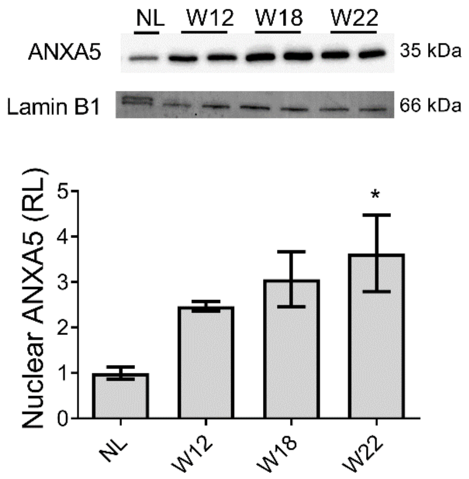

Original blots of Figure 4

General information

Annexin A5 Nuclear. ANXA1 [1:300] NBP1-90162; RRID: AB_11018791
5-second exposure time
Theoretical MW: 35 KDa
Supplier-reported MW: 35-55 KDa
Estimation MW:35.2 KDa

Lamin β1. [1:10000]; Ab16048; RRID: AB_443298
5-min exposure time
Theoretical MW: 66 KDa
Supplier-reported MW: 50-75 KDa
Estimation MW: 66.3 KDa

Original images captured with the Uvitec MINI HD6 photo documentation system.

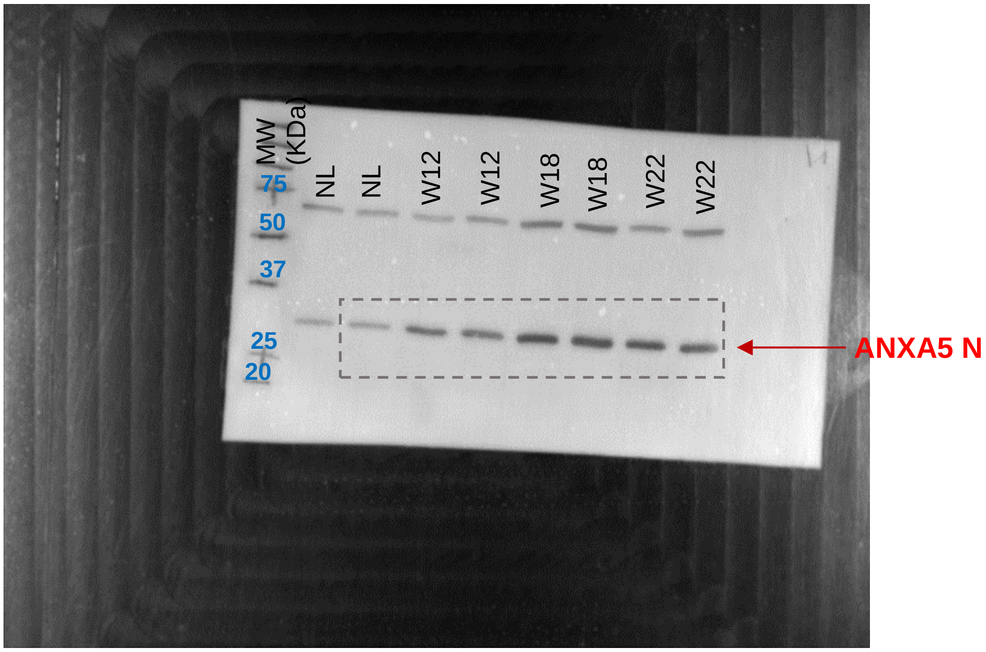

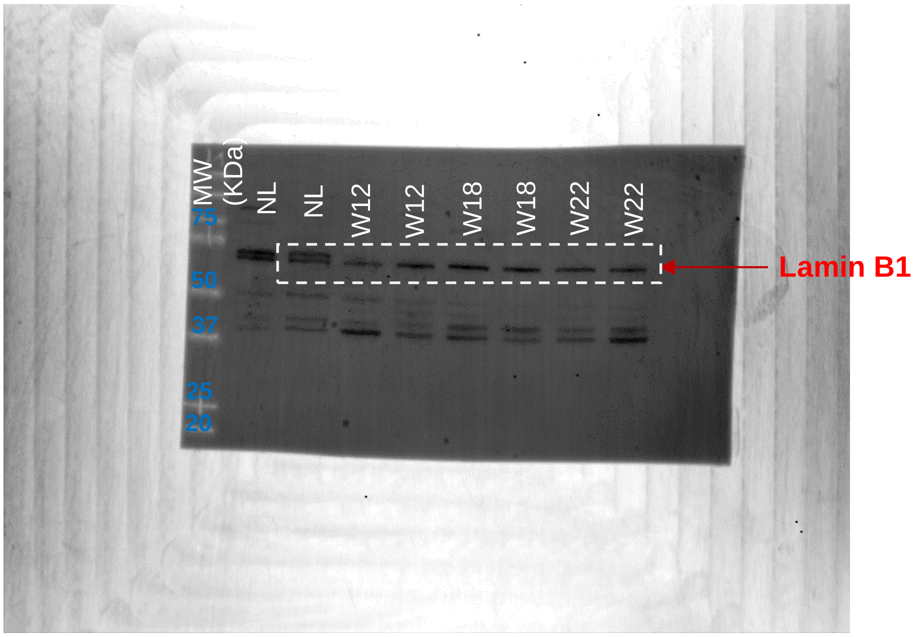

Original blots of Figure 4

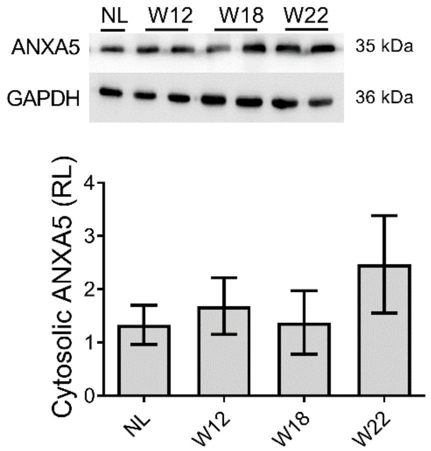

General information

Annexin A5 Cytosolic. ANXA1 [1:300] NBP1-90162; RRID: AB_11018791
5-second exposure time
Theoretical MW: 35 KDa
Supplier-reported MW: 35-55 KDa
Estimation MW:34.7 KDa

GAPDH, [1:10000]; ab181602; RRID: AB_2630358
4-second exposure time
Theoretical MW: 36 KDa
Supplier-reported MW: 25-37 KDa
Estimation MW: 36.6 KDa

Original images captured with the Uvitec MINI HD6 photo documentation system.

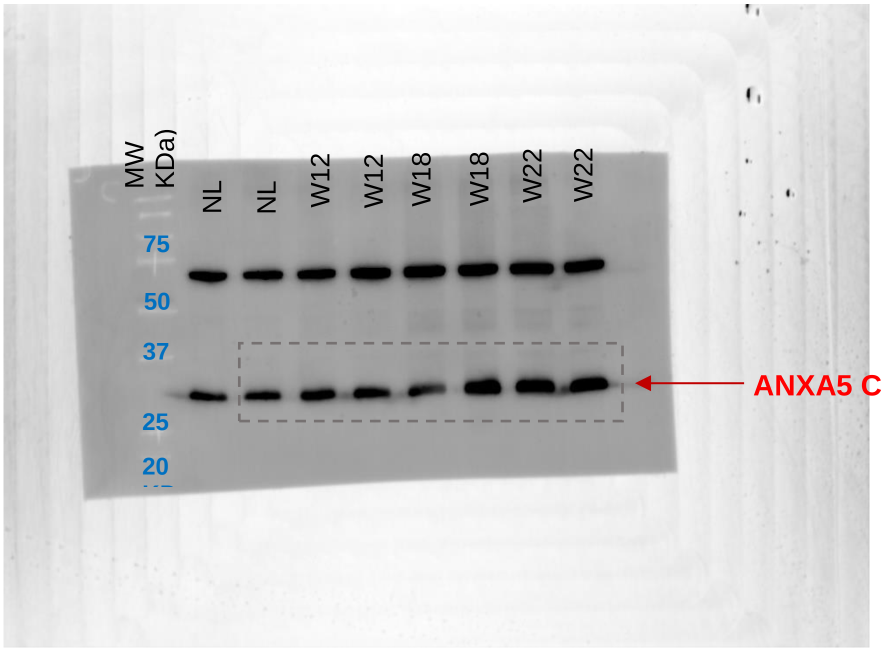

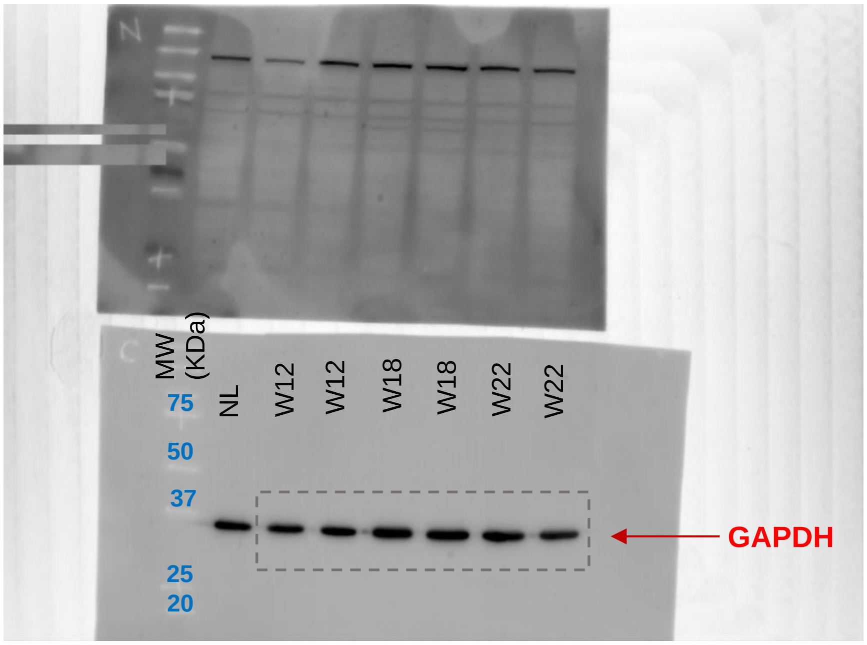

Supplement: Supplementary file 1 — Supplementary Information. [file 41598_2023_34117_MOESM1_ESM.pdf]
